# Highly stable organic photothermal agent based on near-infrared-II fluorophores for tumor treatment

**DOI:** 10.1186/s12951-021-00782-y

**Published:** 2021-02-04

**Authors:** Yunjian Xu, Shiqi Wang, Zhenjiang Chen, Rui Hu, Shaoqiang Li, Yihua Zhao, Liwei Liu, Junle Qu

**Affiliations:** Key Laboratory of Optoelectronic Devices and Systems of Guangdong Province & Ministry of Education, College of Physics and Optoelectronic Engineering Shenzhen University, Shenzhen, 518060 Guangdong Province People’s Republic of China

**Keywords:** Aza-BODIPY, Donor–acceptor–donor structures, Intramolecular photoinduced electron transfer, NIR-II fluorophores, Steric relaxation effect

## Abstract

**Background:**

The aim to develop a highly stable near-infrared (NIR) photoinduced tumor therapy agent stems from its considerable potential for biological application. Due to its long wavelength, biological imaging exhibits a high signal-to-background ratio, deep tissue penetration and maximum permissible light power, which can minimize damage to an organism during photoinduced tumor therapy.

**Results:**

A class of stable NIR-II fluorophores (**NIR998, NIR1028, NIR980, NIR1030**, and **NIR1028-S)** based on aza–boron–dipyrromethene (aza-BODIPY) dyes with donor–acceptor-donor structures have been rationally designed and synthesized by harnessing the steric relaxation effect and intramolecular photoinduced electron transfer (IPET). These fluorophores exhibit an intense range of NIR-II emission, large Stokes shift (≥ 100 nm), excellent photothermal conversion performance, and superior stability against photobleaching. Among the NIR-II fluorophores, **NIR998** possesses better NIR-II emission and photothermal conversion performance. **NIR998** nanoparticles (**NIR998 NPs)** can be encapsulated by liposomes. **NIR998 NPs** show superior stability in the presence of light, heat, and reactive oxygen nitrogen species than that of indocyanine green **NPs**, as well as a higher photothermal conversion ability (η = 50.5%) compared to other photothermal agents. Finally, under the guidance of photothermal imaging, **NIR998 NPs** have been proven to effectively eliminate tumors via their excellent photothermal conversion performance while presenting negligible cytotoxicity.

**Conclusions:**

Utilizing IPET and the steric relaxation effect can effectively induce NIR-II emission of aza-BODIPY dyes. Stable **NIR998 NPs** have excellent photothermal conversion performance and negligible dark cytotoxicity, so they have the potential to act as photothermal agents in biological applications.
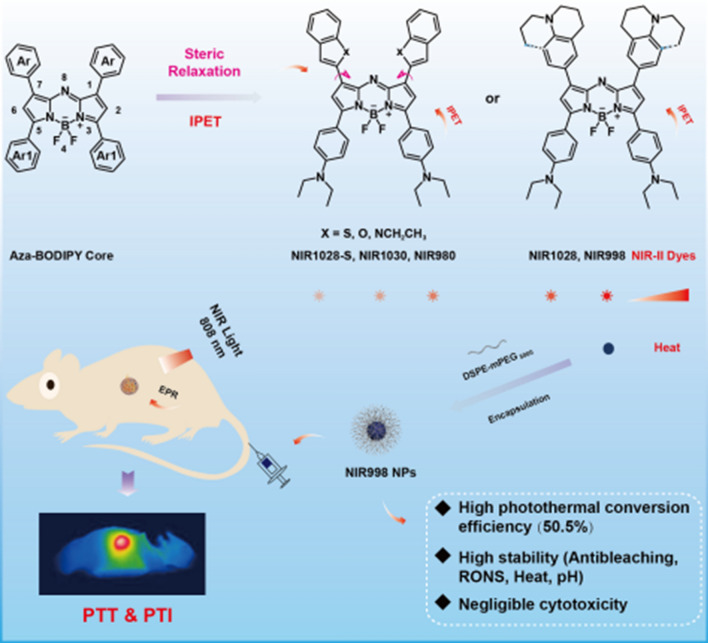

## Background

The development of stable near-infrared (NIR) fluorophores, especially NIR-II fluorophores, has captured considerable interest due to their high-quality imaging with their higher performance and lower health risks. NIR fluorophores emit longer wavelengths to product biological imaging that achieves a high signal-to-background ratio, deep tissue penetration, and maximum permissible light power [1–4]. Thus, photoinduced tumor therapy can minimizes damage to organisms. To date, various NIR-II fluorophores have been developed from various inorganic materials, such as transition-metal sulfide/oxide, rare-earth nanoparticles, and wide-bandgap semiconductors, because their excellent photo-stabilities and photothermal conversion performance had potential applications in biological imaging and tumor therapy [5–7]. However, the poor biodegradability and potential toxicity of inorganic-material-based NIR-II fluorophores have limited their biological application [8–10]. When comparing organic to inorganic, organic-material-based NIR-II fluorophores, especially with small molecules, exhibit excellent advantages, such as an inherent chemical structure and biodegradability, flexible design, absorption wavelength, and low toxicity [10, 11]. Nevertheless, their poor stability and photothermal conversion properties limit their biological applications as flexible NIR-absorbing imaging contrast agents or therapeutic agents [12]. These limitations can produce misguidance, poor therapeutic results, and/or adverse side effect. Therefore, it is a challenge to rationally design and synthesize stable organic NIR-II small molecular fluorophores.

Currently, there are two main methods of constructing NIR-II organic small molecular fluorophores. The first involves changing the heterocyclic substitutions and conjugation length to construct polymethines derivates [13]. The second involves adjusting the versatile donor–acceptor–donor (D–A–D) structure to develop benzo[1,2-c:4,5-c0]bis([1,2,5]thiadiazole) (BBTD) derivates [14]. With the rational design developed by changing the electron-donating properties of substituents and bonding methods, some BBTD-based NIR-II fluorophores have been reported [15]. Although the excellent photophysical and photochemical performance exhibited by these fluorophores justifies their application in biological imaging and tumor therapy, new NIR-II fluorophores with a stable structure and rational design that are capable of facilitating synthesis are needed.

Aza-boron-dipyrromethene (aza-BODIPY) dyes provide many advantages over cyanine derivatives, such as better stability, strong absorbance, and tunable photophysical properties [16]. They have been explored extensively in photoelectric devices, biological imaging and sensing, and tumor therapy [17–19]. The aza-BODIPY mother core skeleton is a typical electron-deficient structure. It can incorporate an electron donor to construct a D–A–D skeleton for distinct enhanced maximal absorption and emission [20], which demonstrates their potential as NIR-II fluorophores for biological imaging and therapy.

**Scheme 1:**
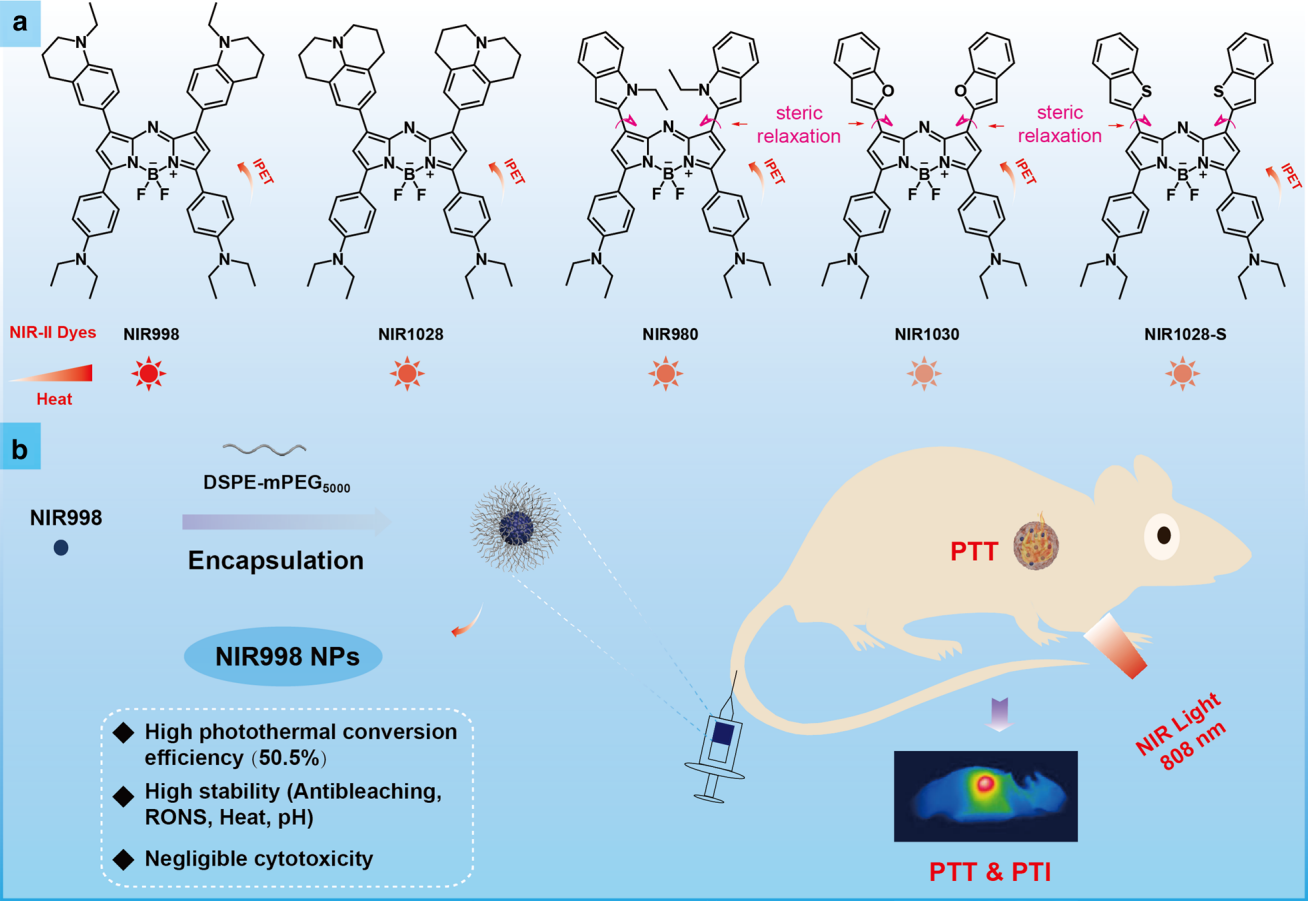
**The machenism of developing NIR-II fluorophores and PTI guided tumor PTT.** **a** IPET and steric relaxation effect for developing stable NIR-II fluorophores based on aza-BODIPYs. **b** Schematic of the preparation of stable NIR998 NPs and their photothermal imaging and therapy

In this work, a series of aza-BODIPY dye-based NIR-II fluorophores (**NIR998, NIR1028, NIR980, NIR1030,** and **NIR1028-S**) were rationally developed via the steric relaxation effect and intramolecular photoinduced electron transfer (IPET) (Scheme 1a). Each exhibits NIR-II emission, a large Stokes shift (≥ 100 nm), and excellent photostability and photothermal conversion performance. Of these NIR-II fluorophores, **NIR998** achieves the best fluorescence emission and photothermal conversion performance, so was selected for encapsulation by liposomes (Scheme 1b). The prepared **NIR998** nanoparticles (**NIR998 NPs**) possessed excellent water solubility and biocompatibility and achieved better resistance to photobleaching, heat and reactive oxygen nitrogen species stability than that of indocyanine green **(ICG) NPs**. The concentration-dependent temperature change and excellent enhanced permeability and retention (EPR) effects of **NIR998 NPs** made them attractive for photothermal imaging of mice (Scheme 1b). Because of their superior capabilities, **NIR998 NPs** have effectively removed ovarian tumors under heat with guidance of photothermal imaging. Furthermore, hematoxylin and eosin (H&E) staining and immunohistochemical analyses have revealed the negligible dark cytotoxicity of **NIR998 NPs**. Therefore, IPET and steric relaxation effect are effectively strategies for exerting NIR-II emission of aza-BODIPY dyes and **NIR998 NPs** with superior stability and high photothermal conversion capabilities have exhibited promising clinical applications as diagnostic reagents.

## Results and discussion

### Design and synthesis of NIR-II fluorophores

The D–A–D system with intramolecular photoinduced electron transfer properties is an effective strategy for developing low-band gap NIR dyes for biological application in vivo [21]. Aza-BODIPY as an inherent photothermal agent was chosen as the research object [22], and aza-BODIPYs with D–A–D structures, especially, with alkyl aniline parts serving as a strong electron donor, exhibit satisfactory NIR-I absorption and emission [23]. In addition, an electron donor with small steric hindrance can also boost the red-shifted absorption and emission capability of aza-BODIPYs core via the steric relaxation effect [24]. Thus, diethylamine, which has a strong donor electronic ability, is introduced at positions 3 and 5 of aza-BODIPYs core, where it achieves the optimal effect of reducing the energy gap for red-shifted absorption and emission [25]. Finally, a series of NIR-II fluorophores with D-A-D’ structures (**NIR998, NIR1028, NIR980, NIR1030,** and **NIR1028-S**) were rationally developed by introducing other alkyl aniline derivatives or aromatic ring skeletons with low steric bulk at positions 2 and 7 of the aza-BODIPYs skeleton.

As shown in Additional file 1: Scheme S1, ketene structures (X-1, X = 1, 2, 3, 4, 5) with high yields (> 90%) were first synthesized using the Claisen–Schmidt reaction between aldehyde derivatives and diethylamino acetophenone under a mild condition. Nitromethane anions were then introduced by an addition reaction, thereby forming X-2. Homodimer of X-2 provided dipyrromethene derivatives (X-3). Finally, NIR-II dyes (**NIR998, NIR1028, NIR980, NIR1030,** and **NIR1028-S**) were obtained by treating X-3 with BF_3_·OEt_2_ in the presence of diisopropylethylamine. They were fully demonstrated by mass spectrometry (MS) and nuclear magnetic resonance (NMR).

### Photophysical Characterization of NIR-II Fluorophores

**Fig. 1:**
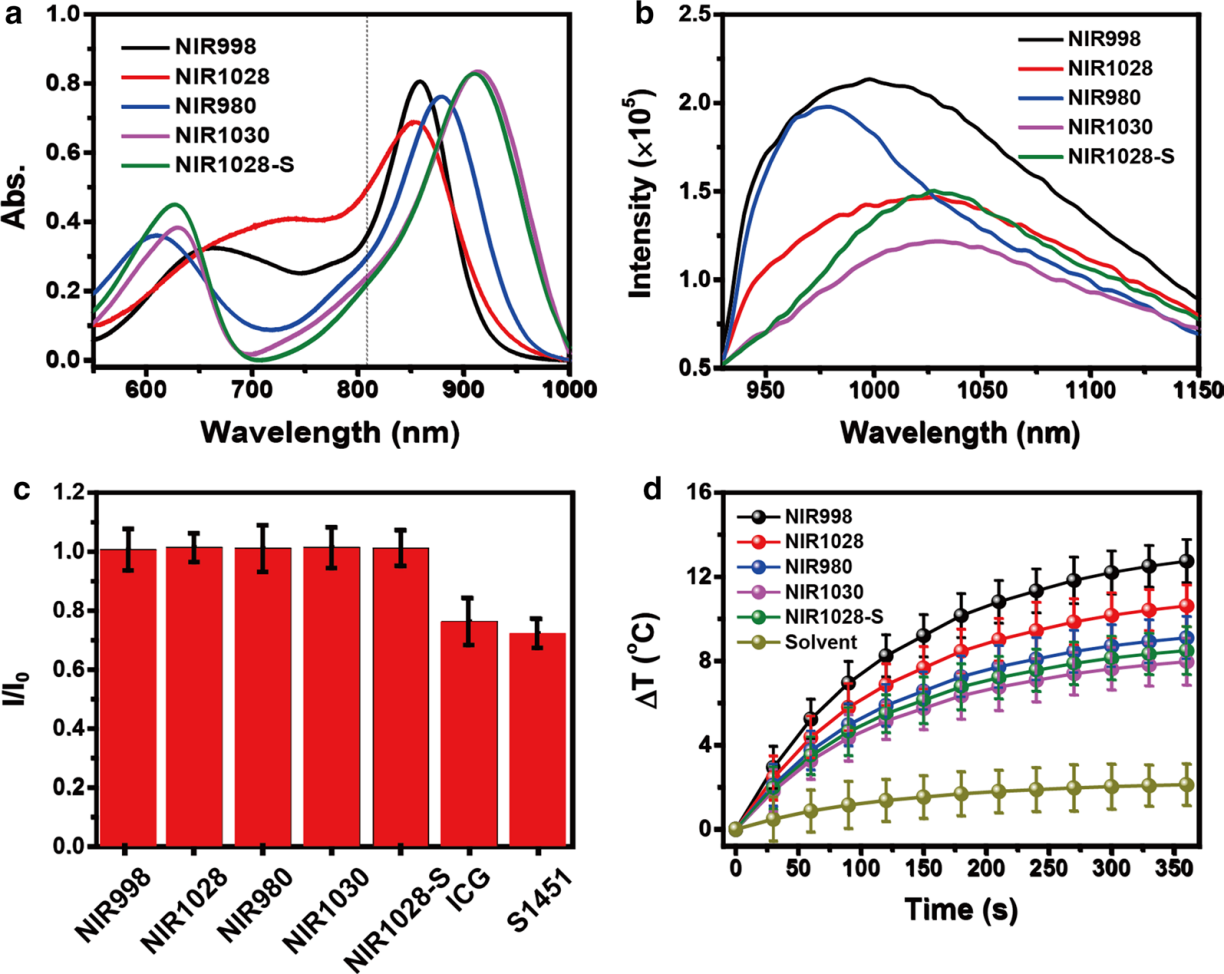
**The photophysical properties of the as-prepared NIR-II fluorophores.** **a**, **b** are absorption and emission spectra of NIR-II dyes (10 μM) in DMSO, respectively. The emission spectra were obtained under irradiation at 808 nm with same absorption. **c** The antiphotobleaching of NIR-II dyes compared with commercial dyes (ICG and S1451) under irradiation (808 nm, 0.2 W cm^−2^, 10 min). I/I_0_ is the ratio of the maximal absorption of dyes after and before irradiation for 10 min, respectively. **d** Temperature changes of NIR-II dyes (10^−5^ M) in DMSO under irradiation (808 nm, 0.5 W cm^−2^, 6 min). The starting temperature is 22 °C

The photophysical properties of free NIR-II dyes were first explored. They showed good dispersity in organic solvents, which indicated outstanding processability (Additional file 1: Fig. S1). Their maximal absorption peak (MAP) exhibited distinct differences in various solvents. When the wavelength of **NIR998**, **NIR1028**, **NIR980**, **NIR1030,** or **NIR1028-S** corresponded to MAP, there was an obvious red shift as 123 nm, 124 nm, 89 nm, 104 nm, or 40 nm, respectively, with the increase in solvent polarity (Additional file 1: Fig. S1), which was due to an intramolecular charge transfer effect [26, 27]. The NIR-II dyes also showed wide absorption in the 500–1000 nm region with the wavelength corresponding to MAP as 859 nm, 853 nm, 880 nm, 913 nm, and 910 nm, respectively, in dimethyl sulfoxide (DMSO) (Fig. 1a). **NIR998, NIR980, NIR1028-S, NIR1028,** and **NIR1030** displayed NIR-II emission with a wavelength corresponding to the maximal emission peak at 998 nm, 980 nm, 1028 nm, 1028 nm, and 1030 nm, respectively (Fig. 1b), which suggests that the construction of a D–A–D’ system with IPET and the steric relaxation effect boost the red-shifted emission within the aza-BODIPY framework and induce the NIR-II emission. Furthermore, the NIR-II dyes displayed reduced luminescence intensity when they shared same absorption at excitation wavelength. They also exhibited a large Stokes shift at 139 nm, 175 nm, 100 nm, 117 nm and 117 nm in DMSO (Fig. 1a, b), respectively, which was due to the IPET [28]. Their intense NIR absorption and emission range were in accordance with the biological window, which indicates that they show promise as imaging contrast agents and therapeutic agents in NIR light-regulated biological applications. The antiphotobleaching property of dyes as imaging contrast agents, probes, or photosensitizers is a crucial index for imaging, sensing and disease treatments [29]. The photobleaching resistance of the NIR-II dyes was then investigated under irradiation (808 nm, 0.2 W cm^−2^) by recording the changes in their maximal absorption. Commercial dyes (ICG and S1451) were used as the control. The absorption spectra of all NIR-II dyes showed slight changes after irradiation for 10 min. In marked contrast, the maximal absorption of commercial dyes such as ICG and S1451 reduced distinctively. For example, after 10 min irradiation, the maximal absorption of ICG and S1451 decreased to 75% and 71%, respectively, of the original value while the remaining NIR-II dyes were great than 97% (Fig. 1c and Additional file 1: Fig. S2), which indicates the better antiphotobleaching capability of the prepared NIR-II dyes. The photothermal conversion properties of NIR-II dyes (10 μM) in DMSO were also explored under irradiation. With continued irradiation, the temperature of the **NIR998, NIR1028, NIR980, NIR1030,** and **NIR1028-S** solutions increased to 12.8 °C, 10.6 °C, 9.1 °C, 8.0 °C and 8.5 °C, respectively (Fig. 1d), while DMSO exhibited a negligible temperature increase under the same condition. The results indicated the excellent photothermal conversion performance of the prepared NIR-II dyes. The relatively better NIR-II emission and photothermal conversion performance of **NIR-998** was chosen for the following test.

### Synthesis and characterization of NIR998 NPs

**Fig. 2:**
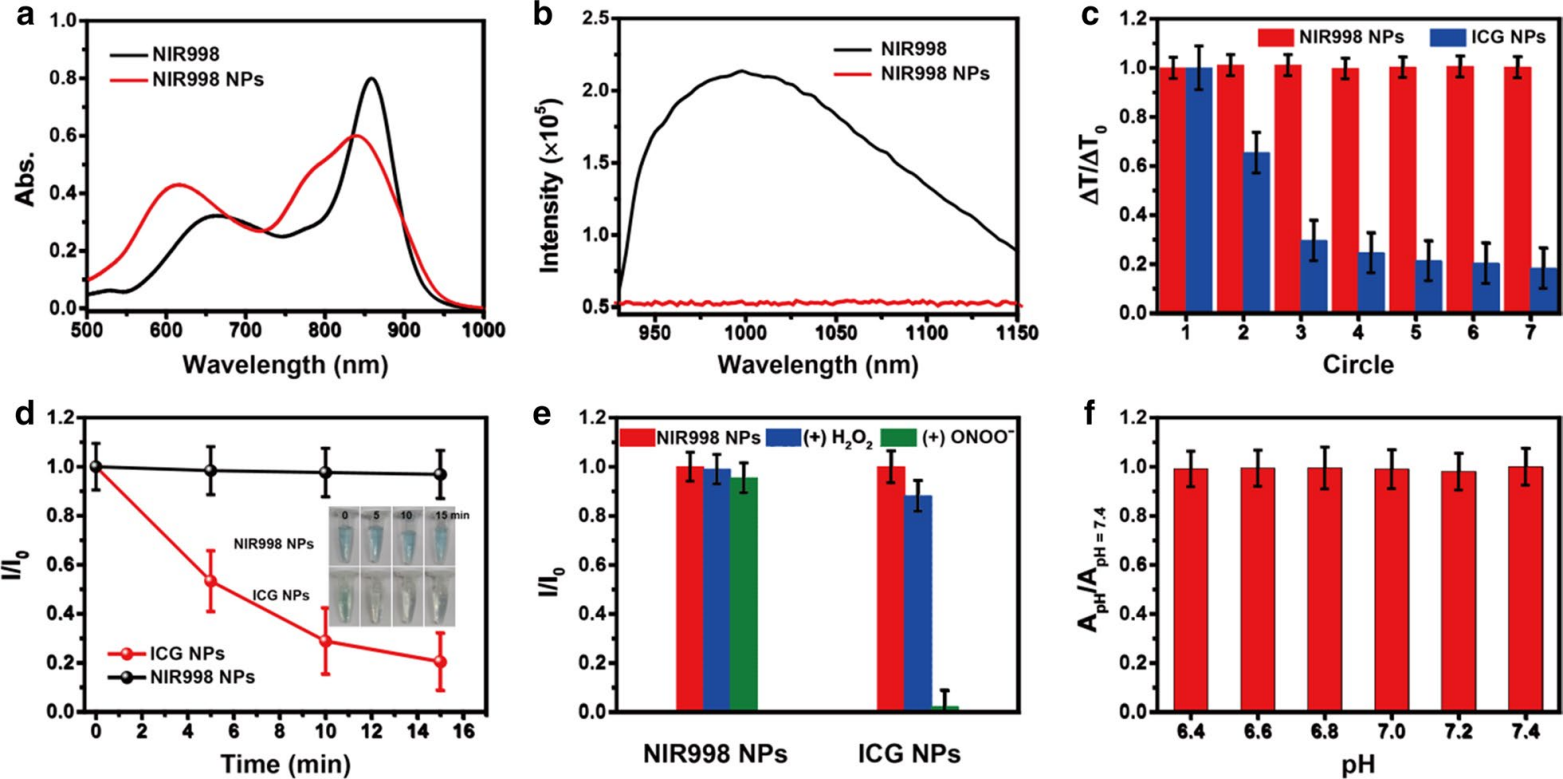
**Stability exploration of NIR998 NPs.** **a**, **b** are absorption and emission spectra of **NIR998** (10 μM) in DMSO and **NIR998 NPs** (10 μM) in PBS, respectively. The emission spectra were acquired under excitation at 808 nm with same absorption. **c** Thermal cycling stability of ICG NPs (20 μM) and **NIR998 NPs** (20 μM) during seven heating and cooling circles (808 nm, 0.5 W cm^−2^). **d** The antiphotobleaching of **NIR998 NPs** (20 μM) and ICG NPs (20 μM). I/I_0_ was the ratio of the maximal absorption of dyes after and before irradiation (808 nm, 0.5 W cm^−2^) with time. Inset: Images of **NIR998 NPs** and **ICG NPs** solutions under various irradiation times, respectively. **e** ONOO^−^ or H_2_O_2_-dependent I/I_0_ change. I/I_0_ was the ratio of the absorption of **NIR998 NPs** plus ONOO^−^ or H_2_O_2_ solutions and **NIR998 NPs** solutions at 859 nm, respectively. **f** pH-dependent A_pH_/A_pH=7.4_ changes. A_pH_ and A_pH=7.4_ represented the absorption of **NIR998 NPs** (859 nm) in PBS with different pH value, respectively

For biological application, water-soluble **NIR998** nanoparticles (**NPs**) with excellent biocompatibility were acquired via encapsulation. The concentration of **NIR998 NPs** was calculated as 400 μM (Additional file 1: Fig. S3). **NIR998 NPs** showed uniform spherical morphology, and their hydrodynamic diameter in phosphate-buffered saline (PBS) (pH = 7.4) was 115 nm, which was larger than the transmission electron microscopy (TEM) results as 75 ± 5 nm due to swelling of the core and liposome in PBS (Additional file 1: Fig. S4). The size of **NIR998 NPs** in PBS contributed to their accumulation at tumor parts due to the EPR effects [30–32]. In addition, the **NIR998 NPs** solutions exhibited similar hydrodynamic diameter for 2 weeks while stored in the refrigerator at 4 °C (Additional file 1: Fig. S5), which indicates that **NIR998 NPs** have excellent water solubility, biocompatibility, and structural stability. **NIR998 NPs** in PBS (pH = 7.4) showed a similar absorption shape, which corresponded to that of **NIR998** in the DMSO solution (Fig. 2a). However, compared to the emission of **NIR998** in the DMSO, the luminescence was severely quenched due to the aggregation of free **NIR998** in the micelles (Fig. 2b). These factors indicated the worse NIR-II fluorescence imaging, which helped to improve the photothermal conversion capability of **NIR998 NPs.** The results were similar to previous works [33–35]. The strong NIR light capture capability and negligible emission of **NIR998 NPs** indicate promising photoacoustic imaging (PAI), photothermal imaging, and tumor photothermal therapy capabilities. The photoacoustic (PA) signal was produced by heat-induced molecular vibration and possessed intense NIR absorption with negligible luminescence, which has been adapted as a nonradiative conversion process for improved photothermal conversion capability [36–38]. The photothermal conversion performance of **NIR998 NPs** solutions was explored in various concentrations. **NIR998 NPs** showed an obvious temperature increase with as the rising concentration or light power (Additional file 1: Fig. S6a, b). In addition, the photothermal conversion efficiency was measured as 50.5% (Additional file 1: Fig. S6c–f), higher than that of most photothermal agents [39–43]. In addition, **NIR998 NPs** (20 μM) solution showed a high temperature increase up to 26 °C under irradiation (808 nm, 0.5 W cm^−2^, 6 min), which helped to effectively suppress tumor growth, indicating that **NIR998 NPs** could serve as promising photothermal agent for tumor photothermal therapy. The **NIR998 NPs** solutions also showed a concentration-dependent PA signal intensity with excellent linearity (R^2^ = 0.9893) (Additional file 1: Fig. S7), which made it possible to quantize the distribution of **NIR998 NPs** in vitro or in vivo.

### Stability exploration of NIR998 NPs

The destroyed agents usually induced misguidance, poor therapeutic efficacy, and/or even serious side effects [44–46]. The photothermal circulation stability, antiphotobleaching properties, pH or reactive oxygen and nitrogen species (RONS) (hydrogen peroxide [ONOO^−^] or peroxynitrite [H_2_O_2_]) resistance were investigated in comparison with **ICG NPs**, which has been verified by the Food and Drug Administration (FDA) for clinical application and demonstrates similar absorption to that of **NIR998 NPs** [47, 48]. The photothermal circulation stability of **NIR998 NPs** and **ICG NPs** in PBS (pH = 7.4) was explored after heating and cooling cycles. The temperature increase (35.9 °C) of **NIR998 NPs** exhibited negligible changes during seven cycles, whereas the temperature changes of **ICG NPs** (27.4 °C) distinctly reduced to 54% (14.8 °C) and 28% (7.6 °C) of the original number after the first and second cycles, respectively (Fig. 2c, Additional file 1: Fig. S8a, b). The results confirmed the better photothermal conversion performance, photothermal circulation stability, and antiphotobleaching of **NIR998 NPs** compared to those of **ICG NPs**. Antiphotobleaching in PBS (pH = 7.4) was further explored under irradiation. As shown in Fig. 2d, Additional file 1: Fig. S8c, d, the **NIR998 NPs** solution displayed negligible changes in colors and absorption spectra, even after 15 min of irradiation, whereas the **ICG NPs** solution exhibited an obviously changed color and decreased maximal absorption. For example, the visible green color disappeared, and only 22% of the **ICG NPs** solution remained after 15 min of irradiation. These results further confirm the superior antiphotobleaching ability of **NIR998 NPs**.

RONS including ONOO^−^ and H_2_O_2_ are considerable important signal molecules and they are over expressed in many diseases [49]. Therefore, it is necessary for therapeutic agents to resist RONS. Then, the RONS-resistant stabilities of **NIR998 NPs** were investigated by recording the absorption spectra with H_2_O_2_, ONOO^−^ or without either. The absorption spectra of **NIR998 NPs** solutions showed negligible change in the H_2_O_2_, and the maximal absorption of **NIR998 NPs** solutions with ONOO^−^ was still greater than 94% of the original number (Fig. 2e, Additional file 1: S8e, f). The maximal absorption of the **ICG NPs** solutions with H_2_O_2_ and ONOO^−^ dramatically decreased to 88% and 7%, respectively. These results confirm the superior resistance of **NIR998 NPs** to H_2_O_2_ and ONOO^−^ compared to that of **ICG NPs**. In addition, the abnormal metabolism of tumor cells induced an acidic extracellular microenvironment (pH = 6.5–6.8) compared with that of blood or normal tissue [50]. Diethylamine parts allow for **NIR998 NPs** to be responsive under acidic conditions. The absorption spectra of **NIR998 NPs** in different pH (6.4–7.4) solutions were then explored. The characteristic absorption spectra of **NIR998 NPs** displayed negligible change in the pH range (Figs. 2f and Additional file 1: Fig. S9), which was attributed to the protection of liposomes. These results demonstrated the superior stability of **NIR998 NPs** under light, heat, RONS and weak acid conditions.

### In vitro cytotoxicity assay of NIR998 NPs

**Fig. 3:**
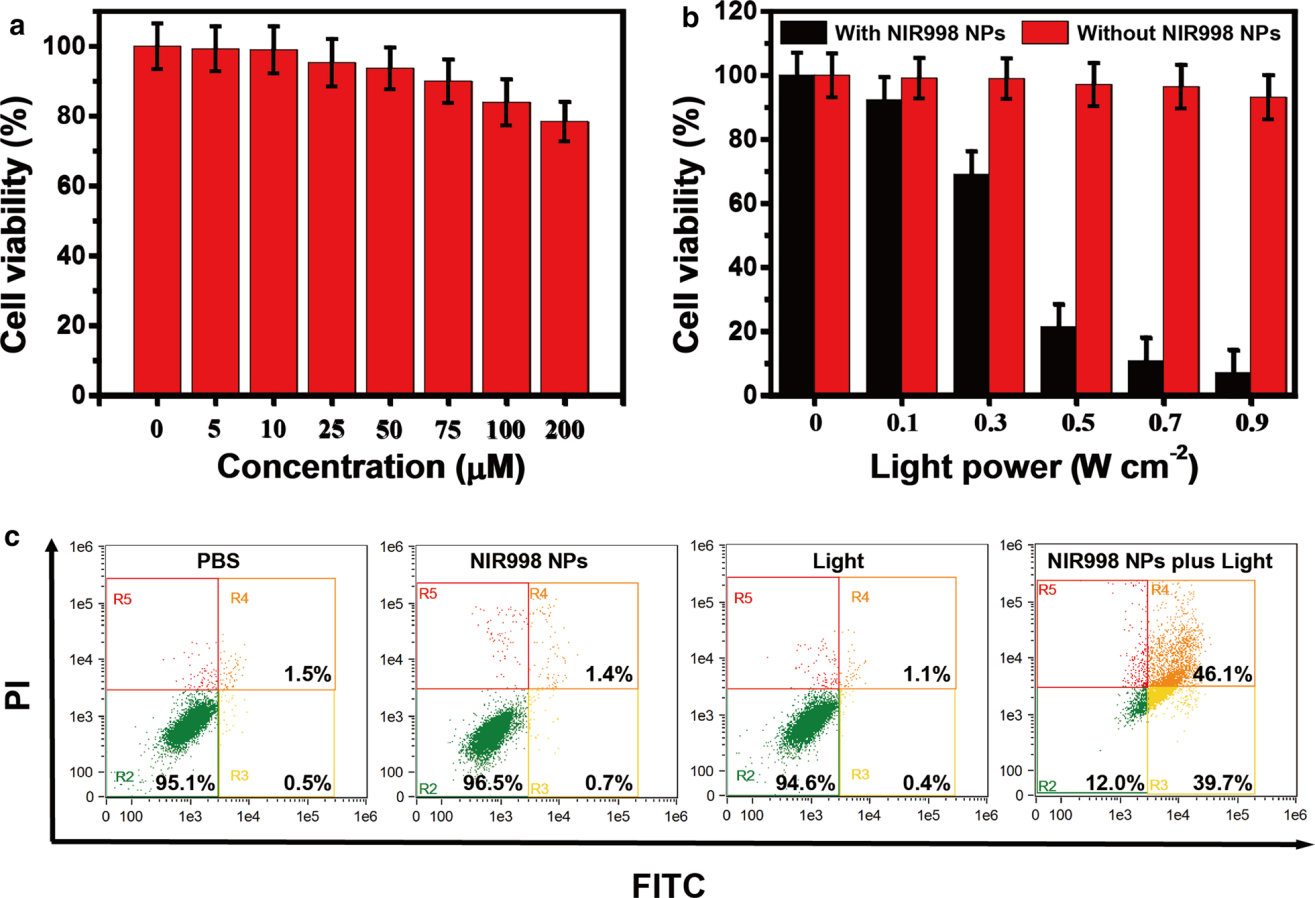
**Cytotoxicity analysis of NIR998 NPs.** **a** The viability of SKOV3 cells treated with **NIR998 NPs** in various concentrations, respectively. **b** The viability of SKOV3 cells after they were incubated with **NIR998 NPs** (20 μM) or **PBS** (pH = 7.4) for 2 h, and then irradiated under various light power, respectively. **c** Flow cytometry quantification of SKOV3 cells treated with PBS, **NIR998 NPs** (20 μM), light irradiation, **NIR998 NPs** plus light irradiation (808 nm, 0.5 W cm^−2^, 6 min), respectively

To explore the photothermal therapy capacity of **NIR998 NPs** in vitro, standard 3-(4,5-dimethylthiazol-2-yl)-2,5-diphenyltetrazolium bromide (MTT) assay was used to assess the dark cytotoxicity of **NIR998 NPs** to SKOV3 cells. **NIR998 NPs** with various concentrations (0–200 μM) were incubated with SKOV3 cells for 24 h. SKOV3 cells treated with various concentrations of **NIR998 NPs** showed high viability, which exhibited a slight decrease as the concentration of **NIR998 NPs** increased. For example, the viabilities were approximately 84% and 78% even at the high concentrations of 100 μM and 200 μM, respectively, (Fig. 3a), which exhibited a slight dark cytotoxicity of **NIR998 NPs** to SKOV3 cells.

To select a reasonable light power for the following tests, SKOV3 cells were incubated with **NIR998 NPs** or **PBS** for 2 h, and then irradiated under various light power, respectively. As shown in Fig. 3b, with the increase of light power, SKOV3 cells without **NIR998 NPs** displayed negligible reduced viabilities, while cells treated with **NIR998 NPs** exhibited rapidly decreased viabilities. For example, under irradiation (0.5 W cm^−2^), cell viabilities were below 25% and above 97% of the original value after incubation in **NIR998 NPs** and PBS, respectively. These results indicate the negligible phototoxicity under 808 nm light and excellent photothermal therapeutic performance of **NIR998 NPs**. Finally, 0.5 W cm^−2^ was selected for subsequent tests.

A flow cytometry experiment was then carried out to further prove the good photothermal effect of **NIR998 NPs**. SKOV3 cells were treated with **NIR998 NPs** (20 μM) plus light irradiation, **NIR998 NPs**, light irradiation, or PBS. Dead cells and apoptotic cells were distinguished by propidium iodide (PI) and Annexin V-FITC, respectively. SKOV3 cells that were treated with **NIR998 NPs**, light irradiation, or PBS displayed high viability (96.5%, 94.6% and 95.1%, respectively) (Fig. 3c), which further confirmed the negligible phototoxicity of light and dark cytotoxicity of **NIR998 NPs** while SKOV3 cells treated with **NIR998 NPs** plus light irradiation showed obviously low cell viability (12.0%) (Fig. 3c), which further demonstrated the excellent photothermal therapeutic effects of **NIR998 NPs** on SKOV3 cells. The same result was also verified by fluorescent imaging of live/dead cell cultivated under the same conditions (Additional file 1: Fig. S10).

### In vivo photothermal imaging of NIR998 NPs

**Fig. 4:**
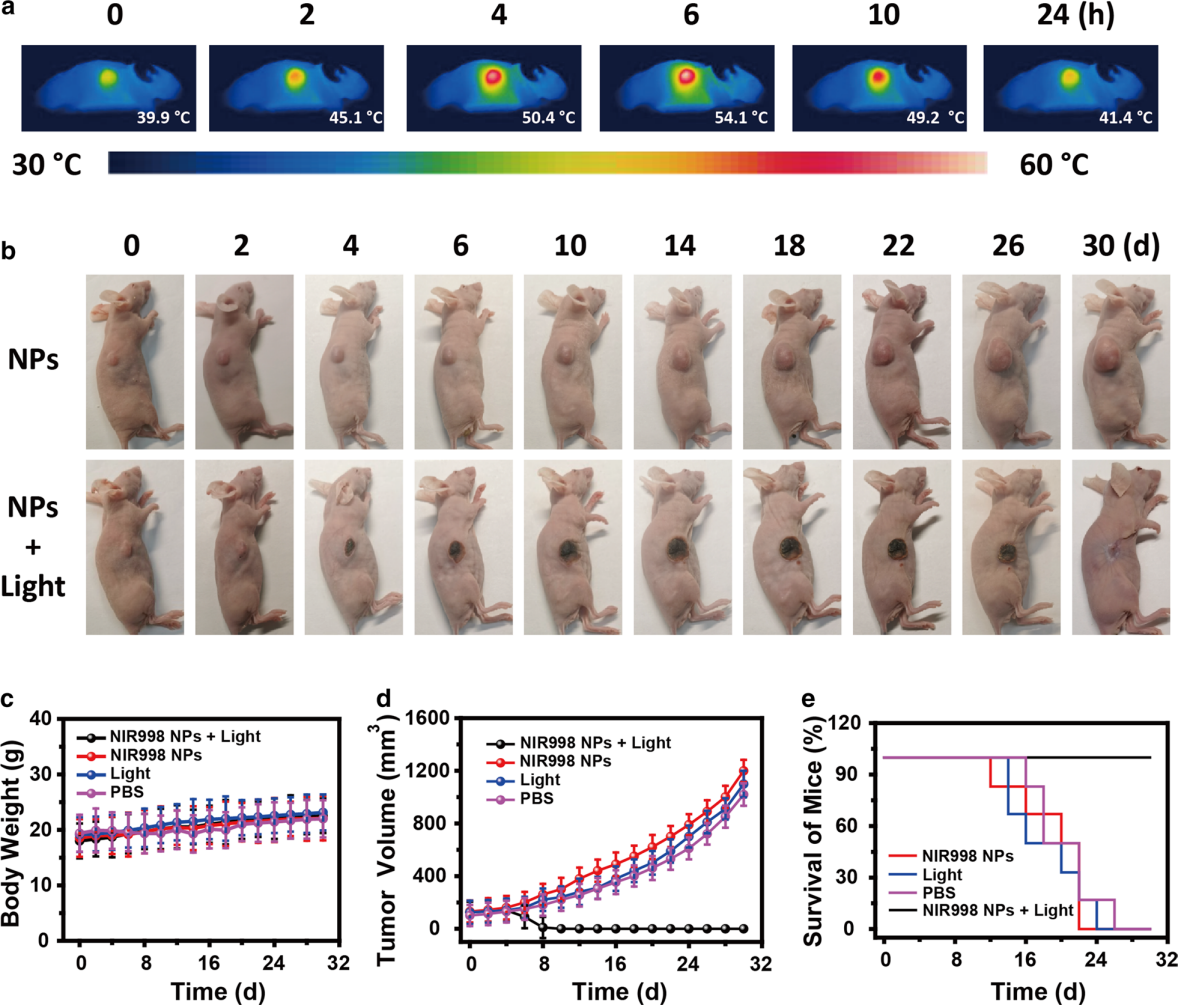
**PTI guided tumor PTT.** **a** is photothermal imaging of mouse with SKOV3 tumor before and after the injection of **NIR998 NPs** with time. The starting temperature of mouse was 36.3 ± 1.5 °C. **b**–**d** are representative images, body weight and tumor size of mice with SKOV3 tumor under different treatments during tumors therapy, respectively. **e** The survival of mice with SKOV3 tumors after various treatments (6 mice per group). The mice were regarded as death after the aggregate tumor burden > 1 cm in diameter

The excellent photothermal therapy of SKOV3 cells motivated us to implement tumor therapy experiments. To explore the optimum time for tumor therapy, which was inspired by the concentration-dependent photothermal conversion performance of **NIR998 NPs**, the photothermal imaging of mice at different times after intravenous injection of **NIR998 NPs** (200 μM, 150 μL) was investigated, due to the accessibility of infrared thermal imaging instruments and easy manipulation. A poor temperature increase (3.6 °C) was observed before injection of **NIR998 NPs** (Fig. 4a). The temperature of the tumor gradually increased over time, and the tumor showed the highest temperature (54.1 °C) after 6 h injection of **NIR998 NPs** (Fig. 4a), which manifested the maximal accumulation of **NIR998 NPs** in the tumor segments due to the excellent passive targeting tumor capacity of **NIR998 NPs** via the EPR effect. The results suggest the excellent accumulation ability of **NIR998 NPs** in tumor after 6 h intravenous injection of **NIR998 NPs**, which was determined to be the optimal time point for next mice tumor therapy.

### Pharmacokinetics study

Pharmacokinetics study of the nanoparticles in major organs, which is an important characteristic to evaluate for tumor therapy, was explored by monitoring the absorption of nanoparticles in serum proteins from mice. The **NIR998 NPs** with excellent stability in the presence of serum proteins showed short terminal elimination half-lives as 0.55 h (Additional file 1: Figs. S11 and S12). The absorption of free **NIR998** obtained from major organs and tumors was further used to assess the biodistribution and accumulation of **NIR998 NPs** after intravenous injection. The major organs and tumors were collected, broken, digested, ultrasonic destroyed after nanoparticles injection in different time. Then, the free molecule was extracted to test their near-infrared absorption. As shown in Additional file 1: Fig. S13, the free molecule obtained from liver, kidney and tumor showed stronger absorption than that of other organs, indicating that the nanoparticles were mainly distributed in liver, kidney and tumor upon injection in the mice for 6 h, and negligible **NIR998 NPs** remained there after 48 h. In addition, the excrement of mice showed negligible NIR absorption compared to that of **NIR998 NPs** (Additional file 1: Fig. S14)**.** The above results confirmed the degradation of **NIR998 NPs** in vivo.

### In vivo PTT

Motivated by the excellent photothermal therapeutic ability of **NIR998 NPs** to SKOV3 cells and effective accumulation of **NIR998 NPs** in tumor segments, photothermal therapy of mice tumors was carried out on the SKOV3 tumor mice model. Twenty-four mice were randomly assigned into four groups. These mice were treated with I) **NIR998 NPs** plus light irradiation; II) **NIR998 NPs** (150 μL, 200 μM); III) PBS + light irradiation (808 nm, 0.5 W cm^−2^, 6 min); and IV) PBS, respectively. Group I served as the experimental group. Group II, III, and IV were used as the control. Mice weight, health condition, and tumor volume were important indexes of tumor therapy results. The mice were monitored at the same time every other day before the next therapy. All mice showed good overall condition, while the weight (~ 20 g) exhibited a similar change trend (Fig. 4b, c, and Additional file 1: Fig. S15). In addition, mice tumors in the control groups exhibited distinct growth over time. The tumor sizes were 10–12 times of the original value after 30 days of treatment (Figs. 4b, d, and Additional file 1: Figs. S15 and S16). Compared with the fast-growing mouse tumors of the control groups, mouse tumors of the experimental group exhibited burned traces before the second therapy, and they were completely removed after the fourth treatment. No new tumors were observed after 30 days (Fig. 4b, d and Additional file 1: Fig. S16). To further value the therapeutic efficiency of the tumors, a survival curve was obtained by regarding mice as dead after the aggregate tumor burden > 1 cm in diameter. After 14 days of treatment, the mice in the control groups died in succession; 24 days later, no mice survived. By marked contrast, no mouse died in the experimental group (Additional file 1: Fig. S16). The above results collectively demonstrate the excellent photothermal therapeutic performance of **NIR998 NPs** to tumors, proving that **NIR998 NPs** show promise as phototherapeutic agents in practical applications.

**Fig. 5:**
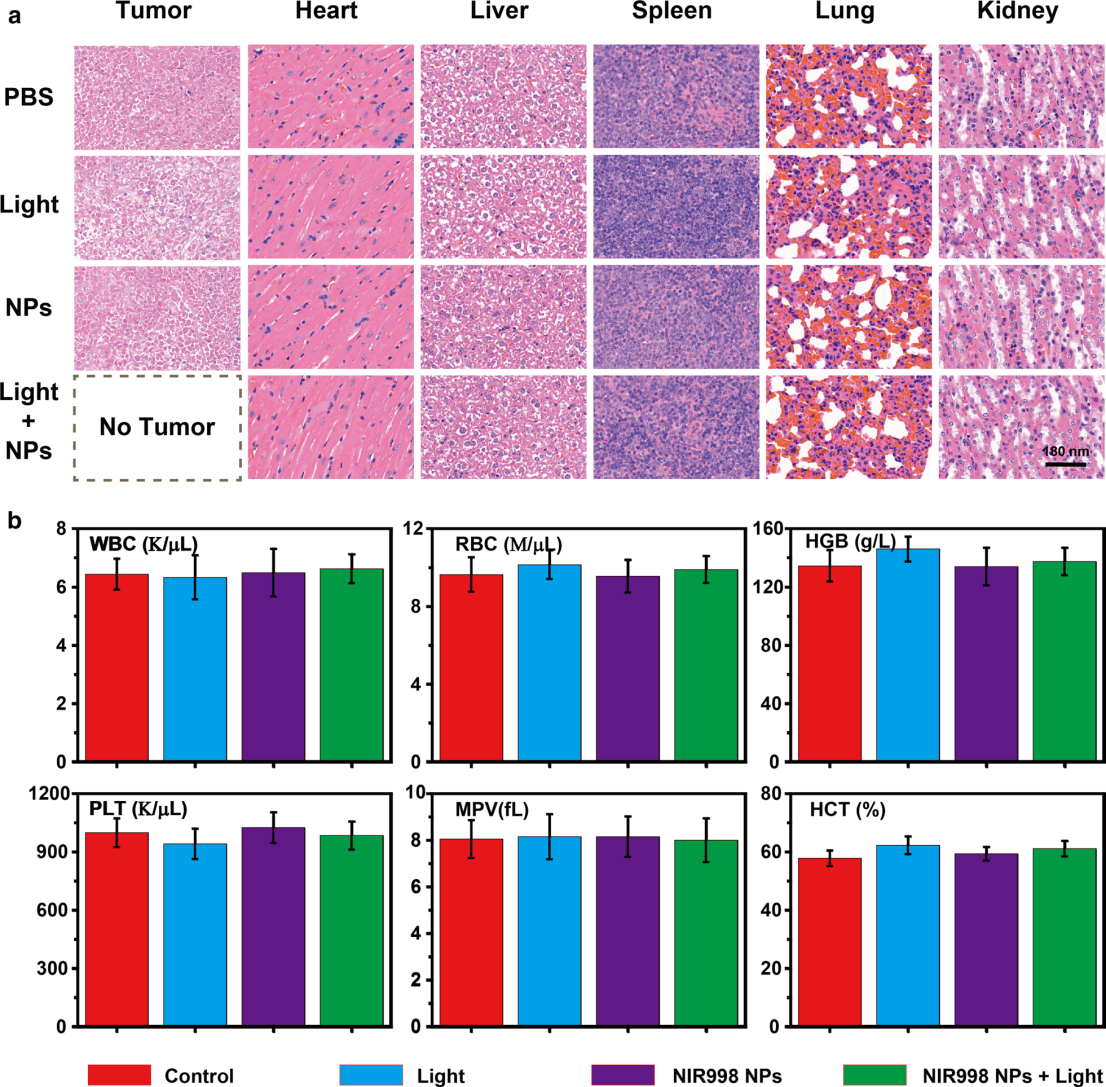
**H&E staining and immunohistochemical analysis.** **a** H&E staining of major organs and tumors acquired from mice treated with **NIR998 NPs** plus Light irradiation, **NIR998 NPs**, Light irradiation and PBS after 30 days treatments, respectively. **b** Blood test parameters in terms of WBC, RBC, HGB, PLT, MPV and HCT of SKOV3 tumor mice treated with **NIR998 NPs** plus light irradiation, **NIR998 NPs**, light irradiation after 30 days treatments, respectively

To further assess the dark cytotoxicity of **NIR998 NPs**, all mice were sacrificed after 30 days of treatments. All major organs were acquired for H&E staining since the nanomaterials were inclined to enrich the reticuloendothelial parts. H&E staining results indicated that all organs and tumors exhibited the same and normal morphological properties with no necrotic areas (Fig. 5a). Blood was also collected for immunohistochemical analyses by comparing the blood indexes, such as the mean platelet volume, hematocrit (HCT), hemoglobin (HGB), and red blood cells (RBC). As is shown in Fig. 5b and Additional file 1: Fig. S17, these important blood indexes exhibited negligible statistical difference among mice undergoing different treatments, which further demonstrates the negligible influence of **NIR998 NPs** or light irradiation on mice normal organs and body weight. The above results fully proved that **NIR998 NPs** are highly biosafe phototherapeutic agents for live mice.

## Conclusion

In summary, a class of aza-BODIPY-dyes-based NIR-II fluorophores (**NIR998, NIR1028, NIR980, NIR1030**, and **NIR1028-S)** with D–A–D’ structures have been rationally developed using the steric relaxation effect and IPET. These fluorophores exhibit an intense range of NIR-II emissions, large Stokes shift (≥ 100 nm) and excellent photothermal conversion performance, and superior stability against photobleaching. Among them, **NIR998** with better NIR-II emission and photothermal conversion capability was used to prepare **NIR998 NPs** by liposome encapsulation. **NIR998 NPs** show better stability than ICG NPs in the presence of light, heat and reactive oxygen nitrogen species, as well as high photothermal conversion performance (η = 50.5%). The excellent photothermal conversion ability and concentration-dependent photoacoustic signal make **NIR998 NPs** excellent photothermal imaging contrast agents and photothermal agents for effectively guiding tumor elimination under mild conditions. The highly efficient tumor photothermal therapeutic performance demonstrated by **NIR998 NPs** through H&E staining and immunohistochemical analyses have revealed the negligible cytotoxicity of **NIR998 NPs** and phototoxicity of light irradiation, which reinforce the potential use of **NIR998 NPs** as diagnostic reagents for clinical applications. Unfortunately, **NIR998 NPs** showed weaker NIR-II emission, which presents a disadvantage to NIR-II fluorescence imaging. Therefore, in future research, we will explore more reasonable strategies for the methods and designs used to develop NIR-II-fluorophore-based NPs with stronger NIR-II emissions.

## Methods

### Materials and characterization

Aldehydes and ketone derivatives were got from Bidepharm. Diethylamine, nitromethane, ethanol, ammonium acetate, and diisopropyl ethylamine were acquired from Macklin. Aldehydes and ketone derivatives were used directly. Other solvents were used without further purification. Nuclear paramagnetic resonance (Bruker Ultra Shield Plus) and mass spectra (Bruker) were used to reveal the chemical structures of compounds. TEM and dynamic light scattering (Nano ZS90) were used for confirming morphology and particle size of nanoparticles, respectively. An ultraviolet–visible light (UV–Vis) spectrophotometer (Cintra 2020) and spectrofluorometer (Horiba Fluoromax-4) were used to record the absorption emission spectra of the samples. Confocal luminescence imaging was conducted using an Olympus IX81 laser scanning confocal microscope. Photothermal images were measured with an NIR thermal imager (FLIR E40). The power density was measured with a VLP-2000 laser power meter.

### Photothermal effect of samples

Temperature changes in the sample solutions were monitored with an infrared thermal imaging system. Temperature changes of the sample solution (300 μL) in different concentrations using different levels of light power were obtained. The processes of temperature increase and decrease in the sample solution (20 μM) with irradiation (808 nm, 0.5 W cm^−2^) and without irradiation were obtained. Finally, the photothermal conversion efficiency of the samples was obtained following the methods of a previous study [23].

### PA signal of NIR998 NPs

A concentration-dependent PA signal of **NIR998 NPs** was obtained using a point-to-point method. The PA signal of **NIR998 NPs** in different concentrations was obtained by monitoring the region of interest, λ_Ex_ = 808 nm.

### Cytotoxicity assay

The dark toxicity assay of **NIR998 NPs** and SKOV3 cells was launched using the standard methyl thiazolyl tetrazolium experiments. SKOV3 cells were treated with **NIR998 NPs** in different concentrations for 24 h. Then, MTT (10 μL/well, 5 mg/mL) was added for further 4 h incubation. Then, 150 μL DMSO was added, and OD570 was measured with an enzyme-linked immunosorbent assay reader. Next, the cell viability was obtained following the method of a previous report.^23^ In addition, SKOV3 cells were treated with PBS or **NIR998 NPs** (20 μM). They were irradiated (808 nm) in various light power conditions for 6 min. Finally, they were treated following the steps used for the above operation.

### In vitro photothermal toxicity of NIR998 NPs

SKOV3 cells were treated with PBS (pH = 7.4); light irradiation (808 nm, 0.5 W cm^−2^, 6 min); **NIR998 NPs** (20 μM); or **NIR998 NPs** plus irradiation. The redundant **NIR998 NPs** were washed with fresh complete medium. Propidium iodide (PI) and annexin V-FITC were used to differentiate between dead cells and apoptotic cells for the subsequent confocal microscopy and flow cytometry analyses.

### Animals and tumor model

Female mice were purchased from Medical Animal Laboratory Center of Guangdong (Permit number: 44007200079864). All in vivo experiments were performed with approval from the Medical Department of Shenzhen University.

### In vivo photothermal imaging exploration

All imaging and therapy exploration of mice were launched in line with standard principles and guidelines. SKOV3-tumor-bearing nude mice with tumor volume of approximately 100 mm^3^ were used for photothermal imaging of mice under irradiation (808 nm, 0.5 W cm^−2^, 6 min) before and after intravenous injection of **NIR998 NPs** (150 μL, 200 μM).

### Pharmacokinetics study

The pharmacokinetics study was conducted by monitoring the NIR absorption of **NIR998 NPs** (150 μL, 200 μM) in serum with time after their intravenous injection in mice. The absorption of free **NIR998** obtained from major organs and tumors was used to assess the biodistribution and accumulation of **NIR998 NPs** after intravenous injection (200 μM, 150 μL) for 6 h. The major organs and tumors were collected, broken, digested, ultrasonic destroyed after nanoparticles injection in different time. Then, the free molecule was extracted to test their near-infrared absorption.

### In vivo photothermal efficacy

SKOV3 tumor-bearing mice with a tumor volume about 100 mm^3^ were assigned into four groups. They were treated with (I) **NIR998 NPs** (150 μL, 200 μM) + light irradiation (808 nm, 0.5 W cm^−2^, 6 min); (II) **NIR998 NPs**; (III) light irradiation; or (IV) PBS. The first group served as experimental groups, and the rest groups worked as control groups. The tumors were irradiated after intravenous injection of **NIR998 NPs** for 6 h. The size of SKOV3 was obtained by V = LW^2^/2, where L and W are the tumor length and width, respectively. The body weight and tumor size of mice were collected every two days for 30 days. At day 30, the major organs and blood of mice were collected for H&E staining and immunohistochemical analyses, respectively.

### Synthesis of NIR-II fluorophores

Synthesis and characterization of **NIR998**. **1–3** (0.31 g, 0.40 mmol) dissolved into the mixture of diisopropylethylamine (4 mL) and dry CH_2_Cl_2_ (20 mL) at 0 °C. Then, BF_3_·OEt_2_ (6.40 mmol) was dropped. They reacted for 8 h at 0 °C. The final reaction solution was quenched by methanol. The precipitate was obtained by vacuum filter. The final blue solid **NIR998** (0.29 g, yield: 91%) was acquired by column chromatography. ^1^H NMR (400 MHz, CDCl_3_) δ(ppm) = 8.08 (d, J = 7.2 Hz, 4H), 7.96 (d, J = 8.4 Hz, 2H), 7.80 (s, 2H), 6.85 (s, 2H), 6.72 (d, J = 7.6 Hz, 4H), 6.65 (d, J = 8.4 Hz, 2H), 3.50–3.31 (m, 16H), 2.85 (t, J = 5.2 Hz, 4H), 2.01 (t, J = 4.8 Hz, 4H), 1.23–1.18 (m, 18H). ^13^C NMR (100 MHz, CDCl_3_) δ(ppm) = 154.34, 147.83, 144.40, 139.94, 130.34, 128.90, 127.87, 121.05, 120.12, 118.23, 113.02, 110.03, 109.23, 47.67, 44.38, 43.40, 27.36, 21.21, 11.75, 10.08. MALDI-TOF–MS m/z: 804.992.

Synthesis and characterization of **NIR1028**. **2–3** (0.31 g, 0.40 mmol) dissolved into the mixture of diisopropylethylamine (4 mL) and dry CH_2_Cl_2_ (20 mL) at 0 °C. Then BF_3_·OEt_2_ (6.40 mmol) was dropped. They reacted for 8 h at 0 °C. The final reaction solution was quenched by methanol. The precipitate was obtained by vacuum filter. The final blue solid **NIR1028** (0.31 g, yield: 93%) was acquired by column chromatography. ^1^H NMR (400 MHz, CDCl_3_) δ(ppm) = 8.05 (d, J = 9.0 Hz, 4H), 7.59 (s, 4H), 6.81 (s, 2H), 6.71 (d, J = 9.0 Hz, 4H), 3.43 (q, J = 6.6 Hz, 8H), 3.24 (t, J = 5.4 Hz, 8H), 2.78 (t, J = 6.6 Hz, 8H), 2.02–1.97 (m, 8H), 1.21 (t, J = 7.2 Hz, 12H). ^13^C NMR (100 MHz, CDCl3) δ(ppm) = 155.15, 148.81, 144.87, 143.31, 141.31, 131.36, 128.18, 121.23, 121.02, 119.35, 114.37, 111.03, 50.12, 44.42, 28.01, 21.96, 12.78. MALDI-TOF–MS m/z: 829.173.

Synthesis and characterization of **NIR980**. **3–3** (0.29 g, 0.40 mmol) dissolved into the mixture of diisopropylethylamine (4 mL) and dry CH_2_Cl_2_ (20 mL) at 0 °C. Then BF_3_·OEt_2_ (6.40 mmol) was dropped. They reacted for 8 h at 0 °C. The final reaction solution was quenched by methanol. The precipitate was obtained by vacuum filter. The final blue solid **NIR980** (0.28 g, yield: 90%) was acquired by column chromatography. ^1^H NMR (400 MHz, CDCl_3_) δ(ppm) = 8.19–8.09 (m, 4H), 7.68–7.60 (m, 2H), 7.44–7.33 (m, 4H), 7.18–7.13 (m, 4H), 7.00–6.96 (m, 2H), 6.83–6.73 (m, 4H), 4.44 (q, J = 13.2, 4H), 3.48 (q, J = 15.6, 8H), 1.43 (t, J = 6.4 Hz, 6H), 1.26 (t, J = 7.2 Hz, 12H). ^13^C NMR (100 MHz, CDCl_3_) δ(ppm) = 155.08, 154.11, 150.05, 148.64, 131.08, 128.47, 122.19, 120.154, 115.21, 116.23, 115.21, 110.13, 107.36, 47.61, 44.60, 15.60, 12.81. MALDI-TOF–MS m/z: 773.271.

Synthesis and characterization of **NIR1030**. **4–3** (0.27 g, 0.40 mmol) dissolved into the mixture of diisopropylethylamine (4 mL) and dry CH_2_Cl_2_ (20 mL) at 0 °C. Then BF_3_·OEt_2_ (6.40 mmol) was dropped. They reacted for 8 h at 0 °C. The final reaction solution was quenched by methanol. The precipitate was obtained by vacuum filter. The final blue solid **NIR1030** (0.28 g, yield: 96%) was acquired by column chromatography. ^1^H NMR (400 MHz, CDCl_3_) δ(ppm) = 8.18 (d, J = 3.6 Hz, 4H), 7.77–7.66 (m, 4H), 7.57 (d, J = 8.0 Hz, 2H), 7.38–7.28 (m, 6H), 6.76 (s, 4H), 3.48 (q, J = 12.8 Hz, 8H), 1.26 (t, J = 7.2 Hz, 12H). ^13^C NMR (100 MHz, CDCl_3_) δ(ppm) = 156.08, 155.11, 150.65, 149.64, 132.08, 129.47, 125.24, 123.19, 121.54, 118.39, 116.23, 111.43, 111.13, 108.36, 44.60, 12.81. MALDI-TOF–MS m/z: 719.242.

Synthesis and characterization of **NIR1028-S**. **5–3** (0.31 g, 0.40 mmol) dissolved into the mixture of diisopropylethylamine (4 mL) and dry CH_2_Cl_2_ (20 mL) at 0 °C. Then BF_3_·OEt_2_ (6.40 mmol) was dropped. They reacted for 8 h at 0 °C. The final reaction solution was quenched by methanol. The precipitate was obtained by vacuum filter. The final blue solid **NIR1028-S** (0.31 g, yield: 93%) was acquired by column chromatography. ^1^H NMR (400 MHz, CDCl_3_) δ(ppm) = 8.32 (s, 2H), 8.16–8.12 (m, 4H), 7.89–7.85 (m, 4H), 7.43–7.35 (m, 4H), 7.10 (s, 2H), 6.77–6.73 (m, 4H), 3.46 (q, J = 7.2 Hz, 8H), 1.24 (t, J = 7.2 Hz, 12H). ^13^C NMR (100 MHz, CDCl_3_) δ(ppm) = 156.00, 149.68, 145.01, 141.01, 140.48, 135.49, 134.43, 132.02, 125.45, 125.05, 124.64, 124.08, 122.21, 118.28, 117.07, 111.43, 44.61, 12.80. MALDI-TOF–MS m/z: 750.264.

The preparation of **NIR998 NPs** (ICG NPs). 20 mg DSPE-mPEG_5000_ dissolved into 8 mL deionized water under sonication (120 W, 2 min). 1.5 mg **NIR998 (ICG)** in 4 mL THF was dropped in above mixture quickly under sonication (120 W, 2 min). The prepared solution was stirred by blowing its surface with argon at 50 ºC overnight. The blue solution was washed with PBS (pH = 7.4) by a centrifugal-filter for three times. The final concentrated blue solution was used for next experiments.

A concentration-dependent absorption of **NIR998** in DMSO was acquired. The linear relationship between concentration and the absorption of **NIR998** at 859 nm (maximal absorption) in DMSO was then obtained. **NIR998 NPs** solution (150 μL) was dried. Then, their absorption in DMSO was got. Finally, according to above linear relationship, the concentration of **NIR998 NPs** solution was obtained as about 400 μM.

## Supplementary Information

**Additional file 1: Scheme S1.** Synthetic routs and chemical structures of NIR-II dyes, respectively. **Fig. S1.** UV−vis absorption spectra of (**a**) NIR998, (**b**) NIR1028, (**c**) NIR980, (**d**) NIR1030 and (**e**) NIR1028-S in different solvents, respectively. The absorption intensity has been normalized. **Fig. S2.** Absorption spectra of NIR-II dyes and commercial dyes (ICG and S1451) in DMSO before and after irradiation of 808 nm at 0.2 W cm^−2^ for 10 min, respectively. **Fig. S3.** (**a**) UV–vis absorption spectra and (**b**) the absorption at 859 nm of NIR998 with various concentrations in DMSO, respectively. **Fig. S4.** (**a**) The transmission electron microscopy photos and (**b**) dynamic light scattering results of NIR998 (10^-5^ M) in PBS (pH = 7.4). **Fig. S5.** (**a**), (**b**), (**c**), (**d**) are dynamic light scattering results of NIR998 (10^-5^ M) in PBS (pH = 7.4) during half a month, respectively. **Fig. S6.** (**a**) Concentrations (808 nm, 6 min) and (**b**) light power dependent (20 μM) temperature rise of NIR998 NPs in PBS (pH = 7.4) in different time, respectively. (**c**) and (**e**) are temperature change of NIR998 NPs (20 μM) solutions and PBS (pH = 7.4) under irradiation (808 nm, 0.5 W cm^−2^), respectively. After the temperature reached to plateau, light irradiation was stopped. (**d**) and (**f**) are time constants of NIR998 NPs (20 μM) solutions and PBS (pH = 7.4) for acquiring photothermal conversion of NIR998 NPs, respectively. **Fig. S7.** Photoacoustic intensity of NIR998 NPs with various concentrations in PBS (pH = 7.4). Inset: photoacoustic intensity images of NIR998 NPs with various concentrations in PBS (pH = 7.4) (λ_Ex_ = 808 nm).  **Fig. S8.** (**a**) and (**b**) are photothermal circulation stability of NIR998 NPs (20 μM) and ICG NPs (20 μM) in PBS (pH = 7.4) under irradiation (808 nm, 0.5 W cm^−2^), respectively. The solutions of samples was irradiated until its temperature reached to plateau. The irradition then stopped. When its temperature decrease to ambient temperature, we then repeated above process for seven times. (**c**) and (**d**) are absorption spectra of NIR998 NPs (20 μM) and ICG NPs (20 μM) in PBS (pH = 7.4) with the absence or presence of H_2_O_2_ (200 μM) or ONOO^−^ (200 μM), respectively. **Fig. S9.** Absorption spectra of NIR998 NPs (20 μM) in PBS (pH = 7.4) with various pH (6.4 – 7.4), respectively. **Fig. S10.** Confocal fluorescence images of SKOV3 cells treated with NIR998 NPs (20 μM) plus light irradiation (808 nm, 0.5 W cm^-2^, 6 min), NIR998 NPs only, light irradiation only or PBS only, respectively. Dead cells and apoptotic cells were distinguished by propidium iodide (PI) and Annexin V-FITC, respectively. **Fig. S11.** (**a**), (**b**), (**c**) are dynamic light scattering results of NIR998 NPs in presence of serum proteins in vitro with time, respectively. **Fig. S12.** (**a**) The absorption spectra of NIR998 NPs in serum after intravenous injection of NIR998 NPs (200 μM, 150 μL) with time. (**b**) Time-dependent-concentrations curves of NIR998 NPs after their intravenous injection in mice. **Fig. S13.** (**a**) and (**b**) are the absorption spectra of free NIR998 obtained from major organs and tumors after intravenous injection of NIR998 NPs (200 μM, 150 μL) for 6 h and 48 h, respectively. **Fig. S14.** The absorption spectrum of NIR998 NPs (10^-5^ M) and excrement of mice in PBS (pH = 7.4) after intravenous injection of NIR998 NPs (200 μM, 150 μL) for 48 h. **Fig. S15.** Representative images of mice with SKOV3 tumor under different treatments during tumors therapy, respectively. **Fig. S16.** SKOV3 tumors of mice with different treatments after 30 days tumor therapy, respectively. **Fig. S17.** Blood test parameters in terms of basophils (BASO), eosnophilshaem (EO), monocyte (MONO), lymphocyte (LYMPH), neutrophile (NEUT) and mean corpuscular hemoglobinregulation (MCH) of SKOV3 tumor mice treated with NIR998 NPs plus light irradiation, NIR998 NPs, light irradiation after 30 days treatments, respectively. Healthy mice act as control. **Figs. S18-S32** are NMR and MALDI-TOF-MS of NIRII dyes.

## Abbreviations

ICG: Indole cyanide green
DMSO: Dimethyl sulfoxide
MTT: Methyl thiazolyl tetrazolium, photothermal therapy
FDA: Food and Drug Administration
NIR: Near-infrared
MS: Mass spectrum
NMR: Nuclear magnetic resonance
