# Supplementary material for: Highly stable organic photothermal agent based on near-infrared-II fluorophores for tumor treatment
Source: J Nanobiotechnology. 2021 Feb 4;19:37. doi: 10.1186/s12951-021-00782-y (PMC7863535; doi:10.1186/s12951-021-00782-y)
Supplement: Supplementary file 1 — Additional file 1: Scheme S1. Synthetic routs and chemical structures of NIR-II dyes, respectively. Fig. S1. UV−vis absorption spectra of (a) NIR998, (b) NIR1028, (c) NIR980, (d) NIR1030 and (e) NIR1028-S in different solvents, respectively. The absorption intensity has been normalized. Fig. S2. Absorption spectra of NIR-II dyes and commercial dyes (ICG and S1451) in DMSO before and after irradiation of 808 nm at 0.2 W cm−2 for 10 min, respectively. Fig. S3. (a) UV–vis absorption spectra and (b) the absorption at 859 nm of NIR998 with various concentrations in DMSO, respectively. Fig. S4. (a) The transmission electron microscopy photos and (b) dynamic light scattering results of NIR998 (10-5 M) in PBS (pH = 7.4). Fig. S5. (a), (b), (c), (d) are dynamic light scattering results of NIR998 (10-5 M) in PBS (pH = 7.4) during half a month, respectively. Fig. S6. (a) Concentrations (808 nm, 6 min) and (b) light power dependent (20 μM) temperature rise of NIR998 NPs in PBS (pH = 7.4) in different time, respectively. (c) and (e) are temperature change of NIR998 NPs (20 μM) solutions and PBS (pH = 7.4) under irradiation (808 nm, 0.5 W cm−2), respectively. After the temperature reached to plateau, light irradiation was stopped. (d) and (f) are time constants of NIR998 NPs (20 μM) solutions and PBS (pH = 7.4) for acquiring photothermal conversion of NIR998 NPs, respectively. Fig. S7. Photoacoustic intensity of NIR998 NPs with various concentrations in PBS (pH = 7.4). Inset: photoacoustic intensity images of NIR998 NPs with various concentrations in PBS (pH = 7.4) (λEx = 808 nm). Fig. S8. (a) and (b) are photothermal circulation stability of NIR998 NPs (20 μM) and ICG NPs (20 μM) in PBS (pH = 7.4) under irradiation (808 nm, 0.5 W cm−2), respectively. The solutions of samples was irradiated until its temperature reached to plateau. The irradition then stopped. When its temperature decrease to ambient temperature, we then repeated above process for seven times. (c) and (d) are abs [file 12951_2021_782_MOESM1_ESM.docx]

**Highly stable organic photothermal agent based on near-infrared-II fluorophores for tumor treatment**

Yunjian Xu^1^, Shiqi Wang^1^, Zhenjiang Chen^1^, Rui Hu^1^, Shaoqiang Li^1^, Yihua Zhao^1^, Liwei Liu^1^* and Junle Qu^1^

^1^Key Laboratory of Optoelectronic Devices and Systems of Guangdong Province & Ministry of Education, College of Physics and Optoelectronic Engineering Shenzhen University, Shenzhen, Guangdong Province, 518060, P. R. China

E-mail: liulw@szu.edu.cn

The manuscript was coauthored by Yunjian Xu (mssn789@szu.edu.cn), Shiqi Wang (2432680983@qq.com), Zhenjiang Chen (127773259@qq.com), Rui Hu (47266743@qq.com), Shaoqiang Li (724566821@qq.com), Yihua Zhao (294596461@qq.com), Liwei Liu (Corresponding author, E-mail: liulw@szu.edu.cn) and Junle Qu (jlqu@szu.edu.cn).

**Contents**

**Part I. Synthesis and characterization of intermediate products**

**Part II. Additional Fig.s**

**Part III. Additional references**

**Part I. Synthesis and characterization of intermediate products**

**Synthesis**.


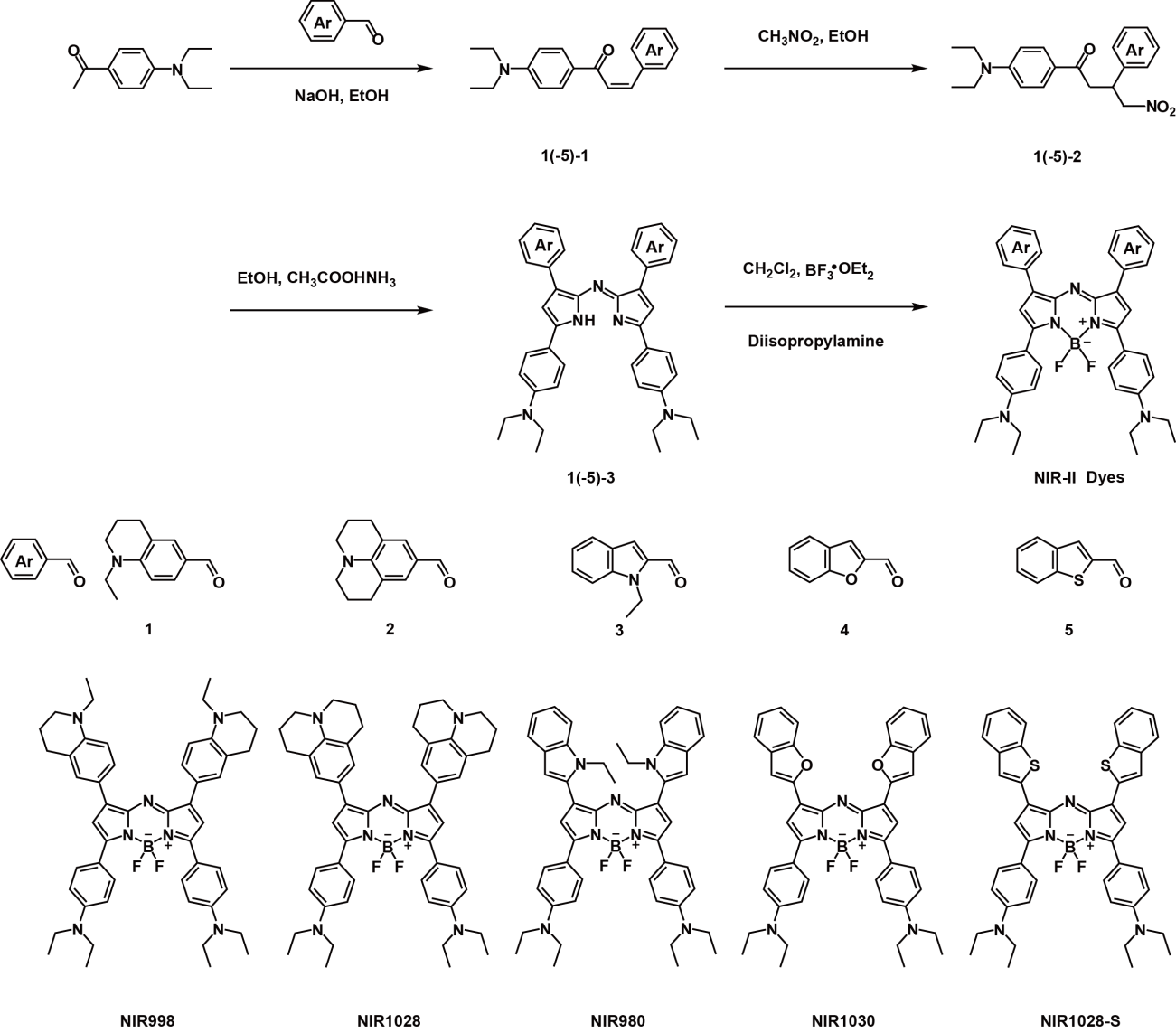


**Scheme S1.** Synthetic routs and chemical structures of **NIR-II** dyes, respectively.

**Synthesis of 1-1.** 1 (1.89 g, 10 mmol) and p-diethylaminoacetophenone (1.91, 10 mmol) was dissolved in EtOH (15 mL), then NaOH solution (1.00 g in 2 mL H_2_O) was then dropped. After reaction at 25 ^o^C for 36 h, 10 mL deionized water was add and the solution was stirred for another 2 h. The final orange solid product was obtained by filtration and desiccation yellow (3.30 g, 91%). ^1^H NMR (400 MHz, CDCl_3_) δ(ppm) = 7.98 (d, J = 7.6 Hz, 2H), 7.72 (d, J = 15.6 Hz, 1H), 7.38 – 7.26 (m, 3H), 6.65 (d, J = 7.6 Hz, 2H), 6.56 (d, J = 8.4 Hz, 1H), 3.44 – 3.34 (m, 6H), 3.31 (t, J = 4.4 Hz, 2H), 2.76 (t, J = 6.4 Hz, 2H), 1.94 (t, J = 5.2 Hz, 2H), 1.22 – 1.13 (m, 9H). ^13^C NMR (100 MHz, CDCl_3_) δ(ppm) = 187.75, 150.74, 146.82, 143.51, 130.79, 129.15, 128.82, 126.07, 122.47, 122.19, 116.12, 110.25, 110.00, 48.54, 45.35, 44.49, 28.12, 21.91, 12.56, 11.03.

**Synthesis of 2-1. 2** (2.01 g, 10 mmol) and p-diethylaminoacetophenone (1.91, 10 mmol) was dissolved in EtOH (15 mL), then NaOH solution (1.00 g in 2 mL H_2_O) was then dropped. After reaction at 25 ^o^C for 36 h, 10 mL deionized water was add and the solution was stirred for another 2 h. The final orange solid product was obtained by filtration and desiccation yellow (3.75 g, 92%). ^1^H NMR (400 MHz, CDCl_3_) δ(ppm) = 7.99 (d, J = 6.8 Hz, 2H), 7.68 (d, J = 15.2 Hz, 1H), 7.33 (d, J = 15.2 Hz, 1H), 7.10 (s, 2H), 6.66 (d, J = 8.0 Hz, 2H), 3.43 (q, J = 5.2 Hz , 4H), 3.23 (t, J = 5.2 Hz, 4H), 2.76 (t, J = 6.4 Hz, 4H), 2.00 – 1.93 (m, 4H), 1.21 (t, J = 6.4 Hz, 6H). ^13^C NMR (100 MHz, CDCl_3_) δ(ppm) = 187.80, 150.72, 144.68, 143.72, 130.81, 127.72, 126.19, 122.36, 121.00, 116.05, 110.26, 49.95, 44.52, 27.71, 21.68, 12.58.

**Synthesis of 3-1. 3** (1.73 g, 10 mmol) and p-diethylaminoacetophenone (1.91, 10 mmol) was dissolved in EtOH (15 mL), then NaOH solution (1.00 g in 2 mL H_2_O) was then dropped. After reaction at 25 ^o^C for 36 h, 10 mL deionized water was add and the solution was stirred for another 2 h. The final orange solid product was obtained by filtration and desiccation yellow (3.26 g, 94%). ^1^H NMR (400 MHz, CDCl_3_) δ(ppm) = 8.04 (d, J = 7.6 Hz, 2H), 7.94 (d, J = 14.8 Hz, 1H), 7.72 (d, J = 15.2 Hz, 1H), 7.65 (d, J = 7.6 Hz, 1H), 7.36 (d, J = 8.0 Hz, 1H), 7.27 (t, J =6.8 Hz, 1H), 7.15 – 7.11 (m, 2H), 6.71 (d, J = 8.0 Hz, 2H), 4.37 (q, J = 6.8 Hz, 2H), 3.47 (q, J = 6.8 Hz, 4H), 1.43 (t, J = 6.8 Hz, 3H), 1.24 (t, J = 6.8 Hz, 6H). ^13^C NMR (100 MHz, CDCl_3_) δ(ppm) = 186.54, 151.24, 137.95, 135.49, 131.09, 129.99, 127.79, 125.29, 123.19, 122.49, 121.31, 120.28, 110.41, 109.57, 102.66, 44.58, 38.02, 15.74, 12.55.

**Synthesis of 4-1. 4** (1.46 g, 10 mmol) and p-diethylaminoacetophenone (1.91, 10 mmol) was dissolved in EtOH (15 mL), then NaOH solution (1.00 g in 2 mL H_2_O) was then dropped. After reaction at 25 ^o^C for 36 h, 10 mL deionized water was add and the solution was stirred for another 2 h. The final orange solid product was obtained by filtration and desiccation yellow (2.97 g, 93%). ^1^H NMR (400 MHz, CDCl_3_) δ(ppm) = 8.04 (d, J = 6.4 Hz, 2H), 7.77 (dd, J = 15.2, 2.4 Hz, 1H), 7.66 (dd, J = 15.6, 3.2 Hz, 1H), 7.59 (d, J = 7.6 Hz, 1H), 7.51 (d, J = 8.0 Hz, 1H), 7.38 – 7.33 (m, 1H), 7.26 – 7.22 (m, 1H), 6.96 (d, J = 1.2 Hz, 1H), 6.68 (d, J = 7.2 Hz, 2H), 3.44 (q, J = 6.4 Hz, 4H), 1.22 (t, J = 5.2 Hz, 6H). ^13^C NMR (100 MHz, CDCl_3_) δ(ppm) = 186.47, 155.40, 153.73, 151.34, 131.28, 128.71, 126.17, 125.14, 123.22, 122.54, 121.63, 111.25, 111.07, 110.40, 44.58, 12.54.

**Synthesis of 5-1. 5** (1.62 g, 10 mmol) and p-diethylaminoacetophenone (1.91, 10 mmol) was dissolved in EtOH (15 mL), then NaOH solution (1.00 g in 2 mL H_2_O) was then dropped. After reaction at 25 ^o^C for 36 h, 10 mL deionized water was add and the solution was stirred for another 2 h. The final orange solid product was obtained by filtration and desiccation yellow (3.22 g, 96%). ^1^H NMR (500 MHz, CDCl_3_) δ(ppm) = 8.15 (d, J = 15.5, 1H), 8.12 – 8.06 (m, 2H), 7.91 – 7.89 (m, 1H), 7.88 – 7.82 (m, 1H), 7.54 (d, J = 15.5 Hz, 1H), 7.49 – 7.41 (m, 2H), 7.36 (s, 1H), 6.80 – 6.76 (m, 2H), 3.55 (q, J = 14.5, 4H), 1.33 (t, J = 7.0 Hz, 6H). ^13^C NMR (125 MHz, CDCl_3_) δ(ppm) = 186.61, 151.46, 141.17, 139.97, 135.31, 131.28, 128.66, 126.08, 125.25, 124.86, 124.37, 123.74, 122.50, 110.55, 44.69, 12.64.

**Synthesis of 1-2****. 1-1** (1.81 g, 5 mmol) was added into the mixture of nitromethane (8 mL), diethylamine (10 mL) and ethanol (10 mL). The mixture reacted under reflux (80 ^o^C) for 72 h. The low boiling point solvent was removed by vacuum pressure. The final product as gray solid (1.86 g, 88%) was obtained via column chromatography (Ethyl acetate: petroleum ether = 1:5). ^1^H NMR (400 MHz, CDCl_3_) δ(ppm) = 7.82 (d, J = 8.8 Hz, 2H), 6.92 (d, J = 8.0 Hz, 1H), 6.81 (s, 1H), 6.60 (d, J = 8.8 Hz, 2H), 6.50 (d, J = 8.4 Hz, 1H), 4.78 (dd, J = 12.4, 6.4 Hz, 1H), 4.59 (dd, J = 11.2, 8.4 Hz, 1H), 4.05 – 3.98 (m, 1H), 3.41 (q, J = 7.2 Hz, 4H), 3.32 – 3.17 (m, 6H), 2.70 (t, J = 6.0 Hz, 2H), 1.95 – 1.89 (m, 2H), 1.19 (t, J = 6.8 Hz, 6H), 1.10 (t, J = 7.2 Hz, 3H). ^13^C NMR (100 MHz, CDCl_3_) δ(ppm) = 195.05, 151.33, 144.42, 130.66, 128.19, 125.92, 123.85, 122.61, 110.59, 110.17, 80.19, 48.27, 45.30, 44.54, 41.11, 39.01, 28.17, 22.15, 12.49, 10.90.

**Synthesis of 2-2. 2-1** (1.87 g, 5 mmol) was added into the mixture of nitromethane (8 mL), diethylamine (10 mL) and ethanol (10 mL). The mixture reacted under reflux (80 ^o^C) for 72 h. The low boiling point solvent was removed by vacuum pressure. The final product as gray solid (1.83 g, 84%) was obtained via column chromatography (Ethyl acetate: petroleum ether = 1:5). ^1^H NMR (400 MHz, CDCl_3_) δ(ppm) = 7.82 (d, J = 9.2 Hz, 2H), 6.66 (s, 2H), 6.61 (d, J = 8.8 Hz, 2H), 4.77 (dd, J = 12.4, 6.4 Hz, 1H), 4.57 (dd, J = 11.6, 9.2 Hz, 1H), 4.02 – 3.90 (m, 1H), 3.42 (q, J = 6.8 Hz, 4H), 3.31 – 3.15 (m, 2H), 3.09 (t, J = 4.2 Hz, 4H), 2.71 (t, J = 6.4 Hz, 4H), 1.90 – 1.89 (m, 4H), 1.20 (t, J = 7.6 Hz, 6H). ^13^C NMR (100 MHz, CDCl_3_) δ(ppm) = 195.05, 151.31, 142.31, 130.64, 126.42, 125.81, 123.83, 121.71, 110.15, 80.11, 49.90, 44.53, 41.15, 39.03, 27.64, 21.98, 12.49.

**Synthesis of 3-2. 3-1** (1.73 g, 5 mmol) was added into the mixture of nitromethane (8 mL), diethylamine (10 mL) and ethanol (10 mL). The mixture reacted under reflux (80 ^o^C) for 72 h. The low boiling point solvent was removed by vacuum pressure. The final product as gray solid (1.81 g, 89%) was obtained via column chromatography (Ethyl acetate: petroleum ether = 1:5). ^1^H NMR (400 MHz, CDCl_3_) δ(ppm) = 7.82 (d, J = 8.8 Hz, 2H), 7.54 (d, J = 8.0 Hz, 1H), 7.33 (d, J = 8.4 Hz, 1H), 7.22 – 7.18 (m, 1H), 7.08 (t, J = 7.6 Hz, 1H), 6.61 (d, J = 7.2 Hz, 2H), 6.38 (s, 1H), 4.86 (dd, J = 12.8, 7.2 Hz, 1H), 4.71 (dd, J = 12.4, 8.0 Hz, 1H), 4.60 – 4.49 (m, 1H), 4.30 (q, J = 6.8 Hz, 2H), 3.46 – 3.27 (m, 6H), 1.44 (t, J = 6.8 Hz, 3H), 1.20 (t, J = 6.8 Hz, 6H). ^13^C NMR (100 MHz, CDCl_3_) δ(ppm) = 193.75, 151.56, 138.36, 136.22, 130.61, 127.78, 123.33, 121.51, 120.33, 119.63, 110.22, 109.46, 98.67, 78.49, 44.55, 41.01, 37.96, 30.86, 15.42, 12.44.

**Synthesis of 4-2. 4-1** (1.60 g, 5 mmol) was added into the mixture of nitromethane (8 mL), diethylamine (10 mL) and ethanol (10 mL). The mixture reacted under reflux (80 ^o^C) for 72 h. The low boiling point solvent was removed by vacuum pressure. The final product as gray solid (1.54 g, 81%) was obtained via column chromatography (Ethyl acetate: petroleum ether = 1:5). ^1^H NMR (400 MHz, CDCl_3_) δ(ppm) = 7.85 (d, J = 7.6 Hz, 2H), 7.48 (d, J = 7.6 Hz, 1H), 7.42 (d, J = 8.0 Hz, 1H), 7.26 – 7.17 (m, 2H), 6.64 – 6.59 (m, 3H), 4.94 – 4.82 (m, 2H), 4.50 – 4.44 (m, 1H), 3.53 – 3.36 (m, 6H), 1.20 (t, J = 6.4 Hz, 6H). ^13^C NMR (100 MHz, CDCl_3_) δ(ppm) = 193.57, 155.51, 154.75, 130.68, 128.18, 124.12, 122.87, 120.97, 111.22, 110.68, 104.09, 44.67, 37.90, 33.87, 12.44.

**Synthesis of 5-2. 5-1** (1.68 g, 5 mmol) was added into the mixture of nitromethane (8 mL), diethylamine (10 mL) and ethanol (10 mL). The mixture reacted under reflux (80 ^o^C) for 72 h. The low boiling point solvent was removed by vacuum pressure. The final product as gray solid (1.57 g, 85%) was obtained via column chromatography (Ethyl acetate: petroleum ether = 1:5). ^1^H NMR (500 MHz, CDCl_3_) δ(ppm) = 7.84 (d, J = 8.5 Hz, 2H), 7.76 (d, J = 7.5 Hz, 1H), 7.68 (d, J = 7.5 Hz, 1H), 7.34 – 7.28 (m, 2H), 7.19 (s, 1H), 6.62 (d, J = 9.0 Hz, 2H), 4.92 (dd, J = 12.5, 5.5 Hz, 1H), 4.77 (dd, J = 12.5, 8.5 Hz, 1H), 4.65 – 4.56 (m, 1H), 3.53 – 3.35 (m, 6H), 1.20 (t, J = 7.0 Hz, 6H). ^13^C NMR (125 MHz, CDCl_3_) δ(ppm) = 193.54, 143.43, 139.51, 139.08, 130.75, 124.40, 123.51, 122.26, 110.37, 79.57, 44.62, 41.02, 35.91, 12.46.

**Synthesis of 1-3. 1-2** (1.27 g, 3 mmol) and ammonium acetate (3.00 g, 40 mmol) reacted in n-butanol (50 mL) under reflux (120 ^o^C) for 48 h. The solvent was removed under vacuum pressure and the solid residue was filtered and washed with cool ethanol (3×20 mL). **1-3** was applied directly for following synthesis.

**Synthesis of 2-3. 2-2** (1.31 g, 3 mmol) and ammonium acetate (3.00 g, 40 mmol) reacted in n-butanol (50 mL) under reflux (120 ^o^C) for 48 h. The solvent was removed under vacuum pressure and the solid residue was filtered and washed with cool ethanol (3×20 mL). **2-3** was applied directly for following synthesis.

**Synthesis of 3-3. 3-2** (1.22 g, 3 mmol) and ammonium acetate (3.00 g, 40 mmol) reacted in n-butanol (50 mL) under reflux (120 ^o^C) for 48 h. The solvent was removed under vacuum pressure and the solid residue was filtered and washed with cool ethanol (3×20 mL). **3-3** was applied directly for following synthesis.

**Synthesis of 4-3. 4-2** (1.14g, 3 mmol) and ammonium acetate (3.00 g, 40 mmol) reacted in n-butanol (50 mL) under reflux (120 ^o^C) for 48 h. The solvent was removed under vacuum pressure and the solid residue was filtered and washed with cool ethanol (3×20 mL). **4-3** was applied directly for following synthesis.

**Synthesis of 5-3. 5-2** (1.19 g, 3 mmol) and ammonium acetate (3.00 g, 40 mmol) reacted in n-butanol (50 mL) under reflux (120 ^o^C) for 48 h. The solvent was removed under vacuum pressure and the solid residue was filtered and washed with cool ethanol (3×20 mL). **5-3** was applied directly for following synthesis.

**Part II. Supplemental Fig.s**

**
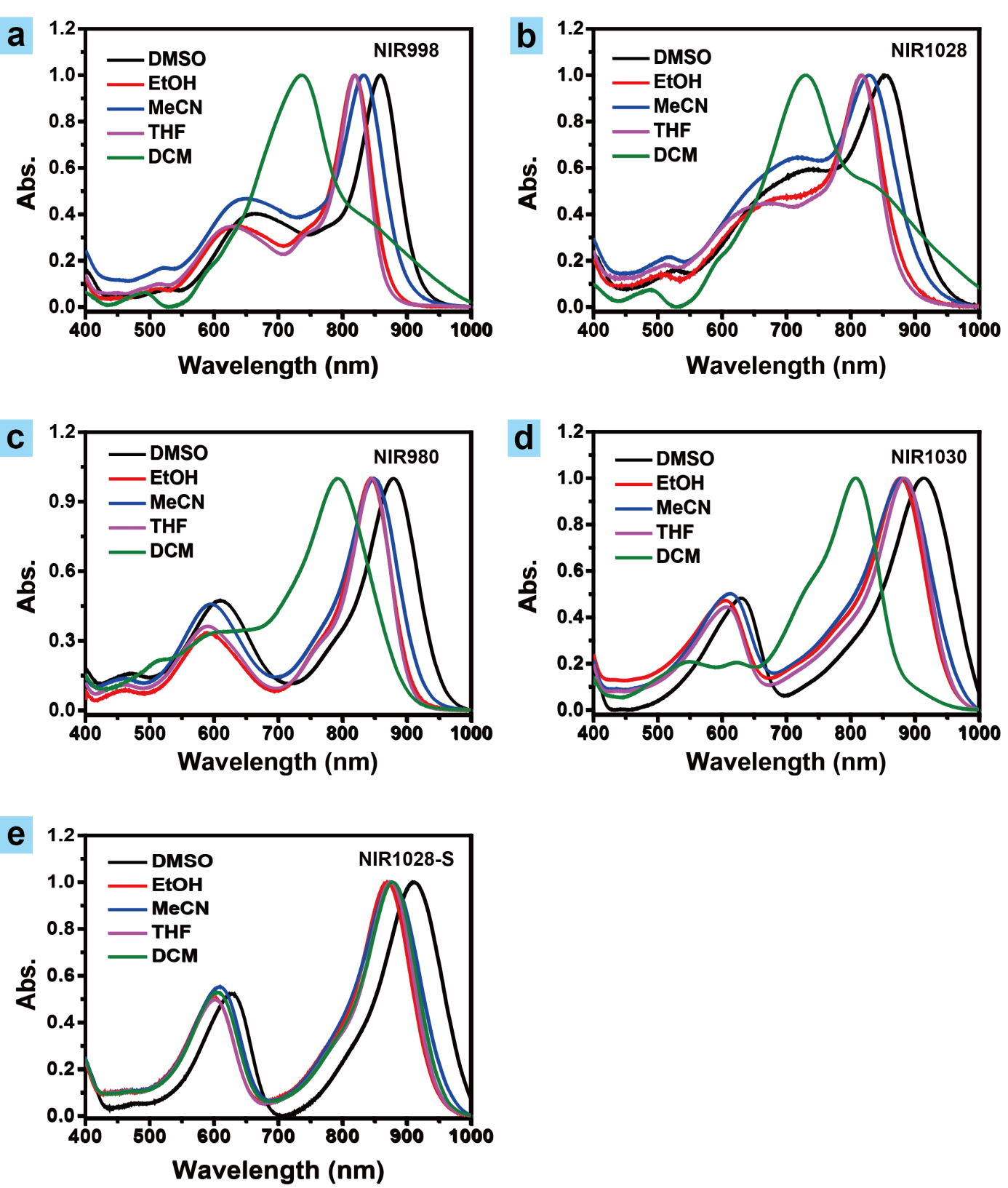
**

**Fig. S1.** UV−vis absorption spectra of (a) **NIR998**, (b) **NIR1028**, (c) **NIR980**, (d) **NIR1030** and (e) **NIR1028-S** in different solvents, respectively. The absorption have been normalized.


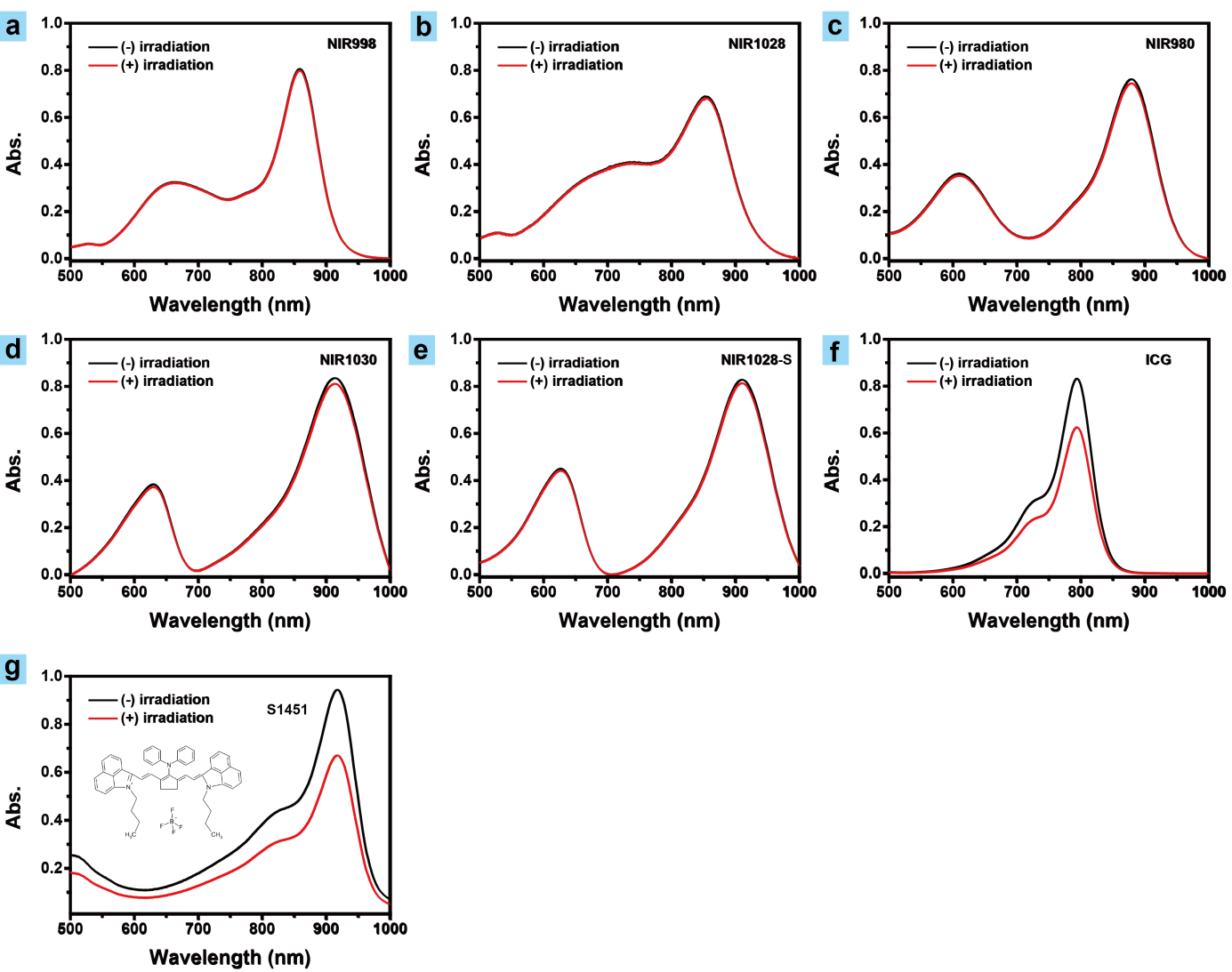


**Fig. S2.** Absorption spectra of **NIR-II** dyes and commercial dyes (ICG and S1451) in DMSO before and after irradiation of 808 nm at 0.2 W cm^−2^ for 10 min, respectively.

**
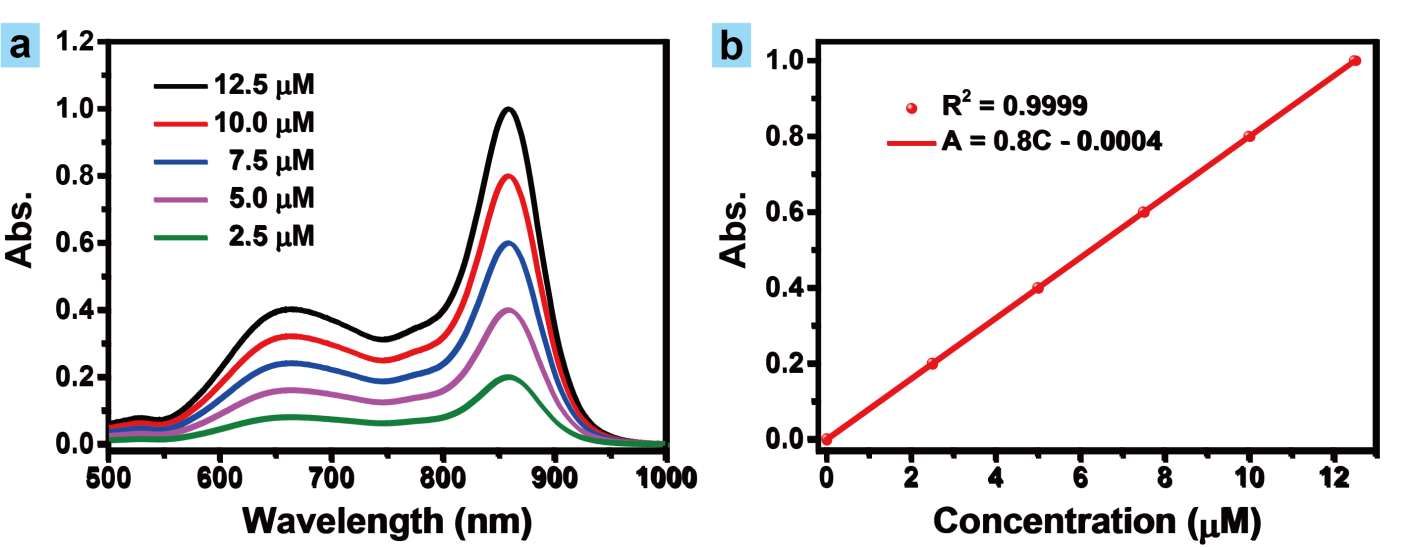
**

**Fig. S3.** (a) UV–vis absorption spectra and (b) the absorption at 859 nm of **NIR998** with various concentrations in DMSO, respectively.


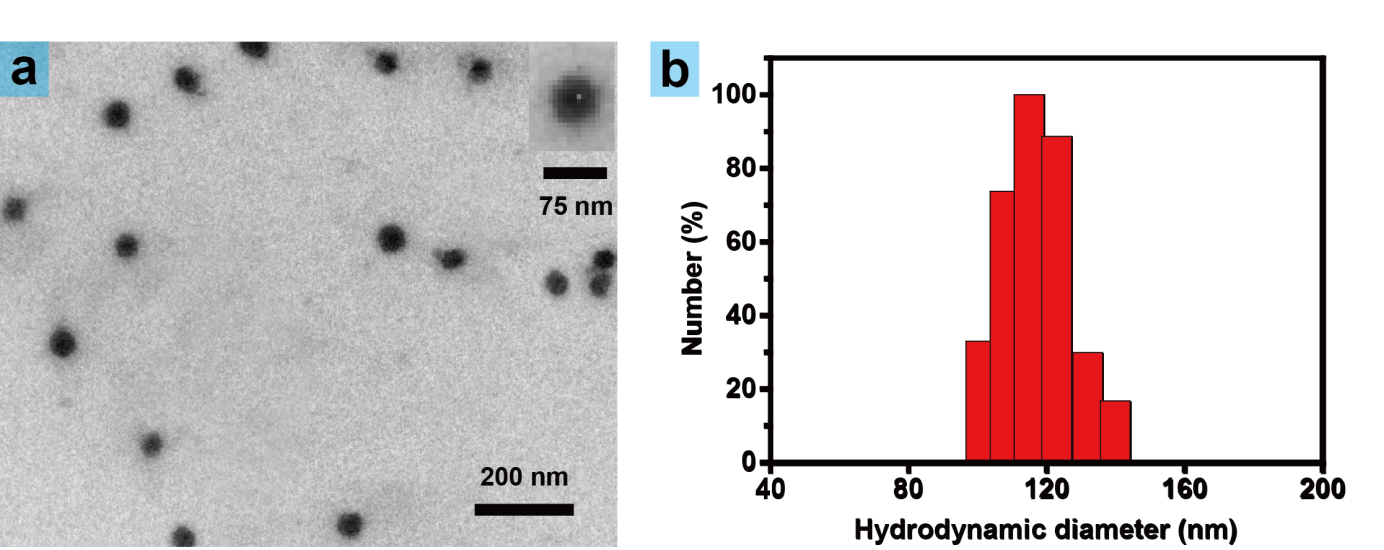


**Fig. S4.** (a) The transmission electron microscopy photos and (b) dynamic light scattering results of **NIR998** (10^-5^ M) in PBS (pH = 7.4).


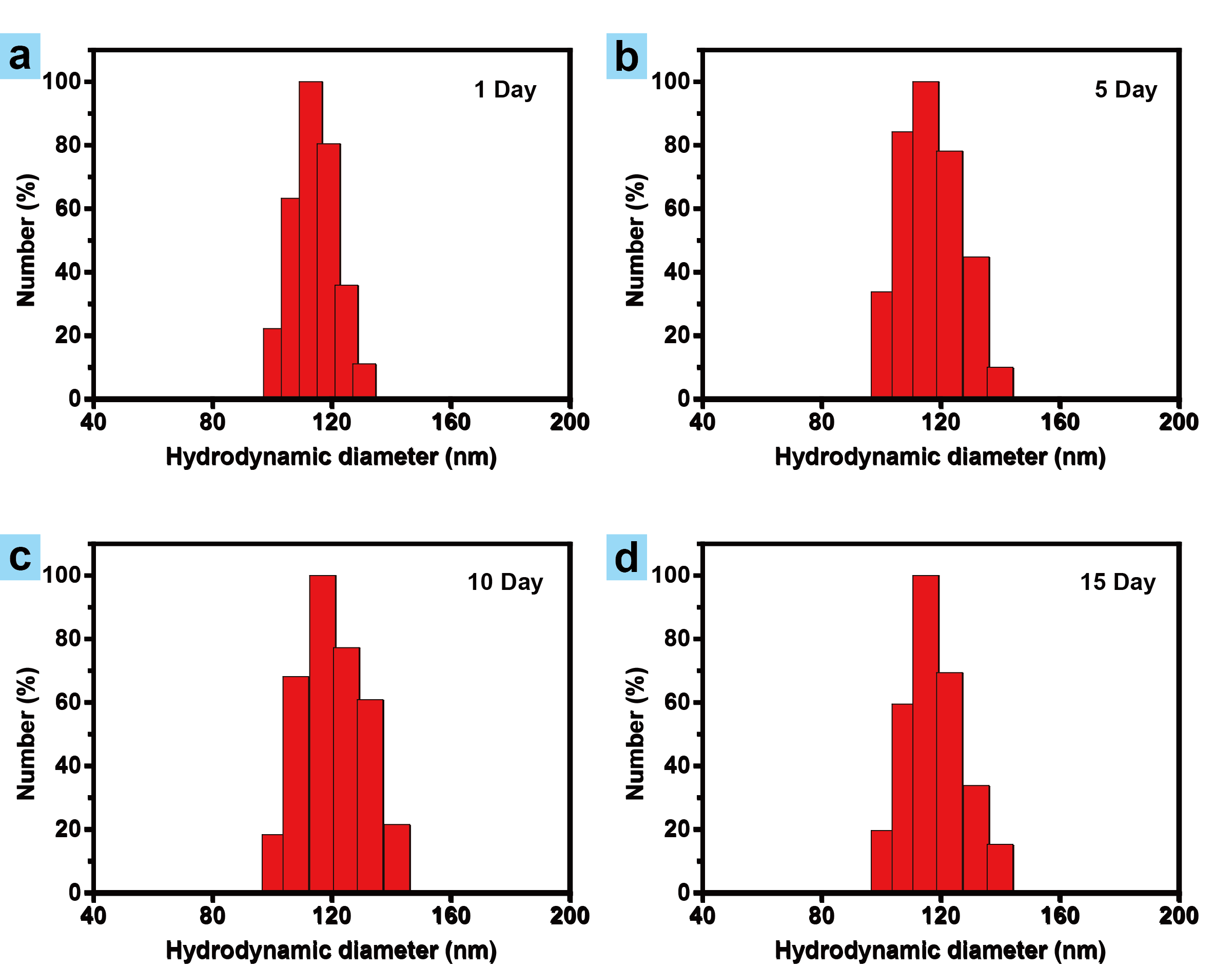


**Fig. S5.** (a), (b), (c), (d) are dynamic light scattering results of **NIR998** (10^-5^ M) in PBS (pH = 7.4) during half a month, respectively.

**
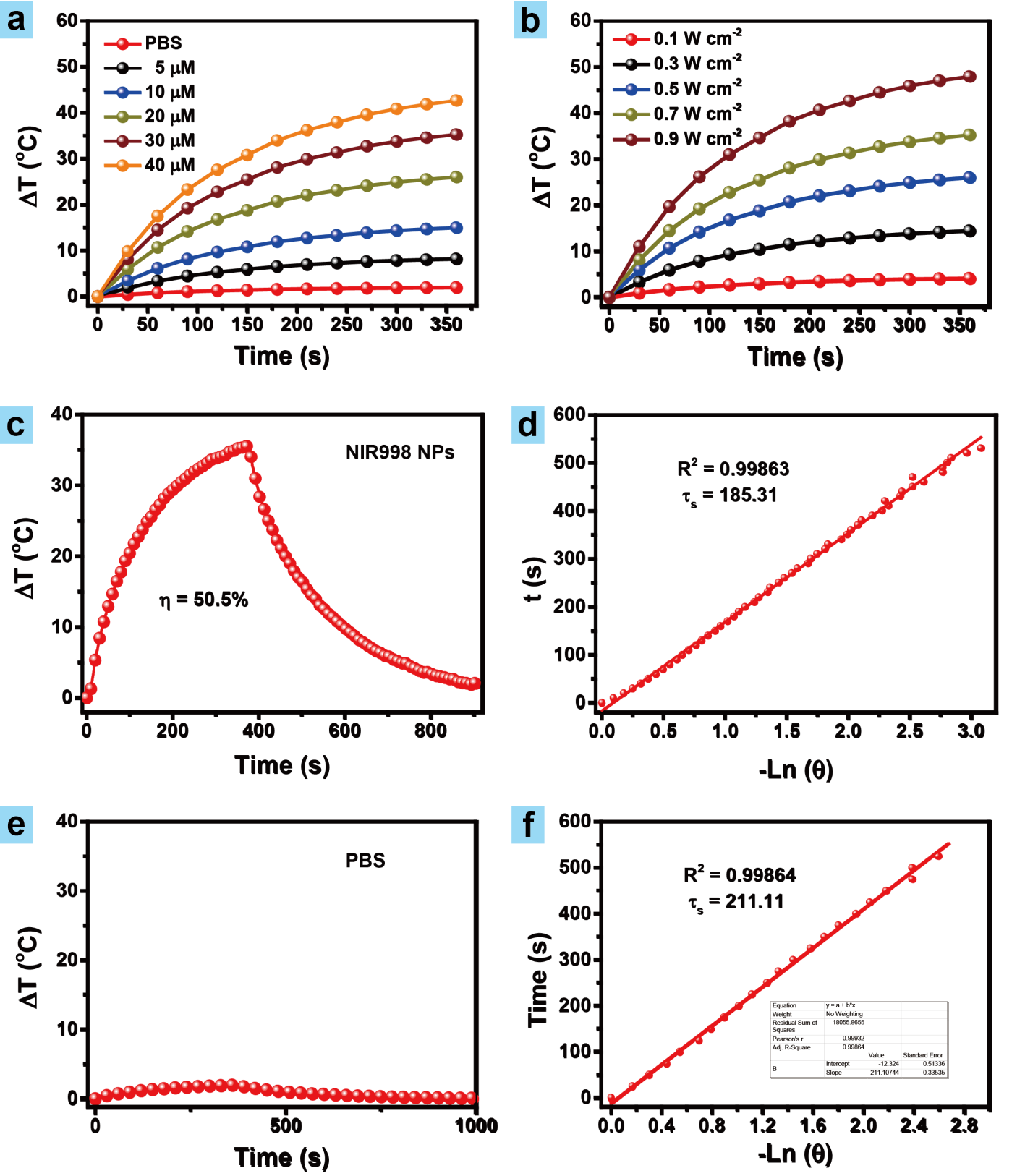
**

**Fig. S6.** (a) Concentrations (808 nm, 6 min) and (b) light power dependent (20 μM) temperature rise of **NIR998 NPs** in PBS (pH = 7.4) in different time, respectively. (c) and (e) are temperature change of **NIR998 NPs** (20 μM) solutions and PBS (pH = 7.4) under irradiation (808 nm, 0.5 W cm^−2^), respectively. After the temperature reached to plateau, light irradiation was stopped. (d) and (f) are time constants of **NIR998 NPs** (20 μM) solutions and PBS (pH = 7.4) for acquiring photothermal conversion of **NIR998 NPs**, respectively.


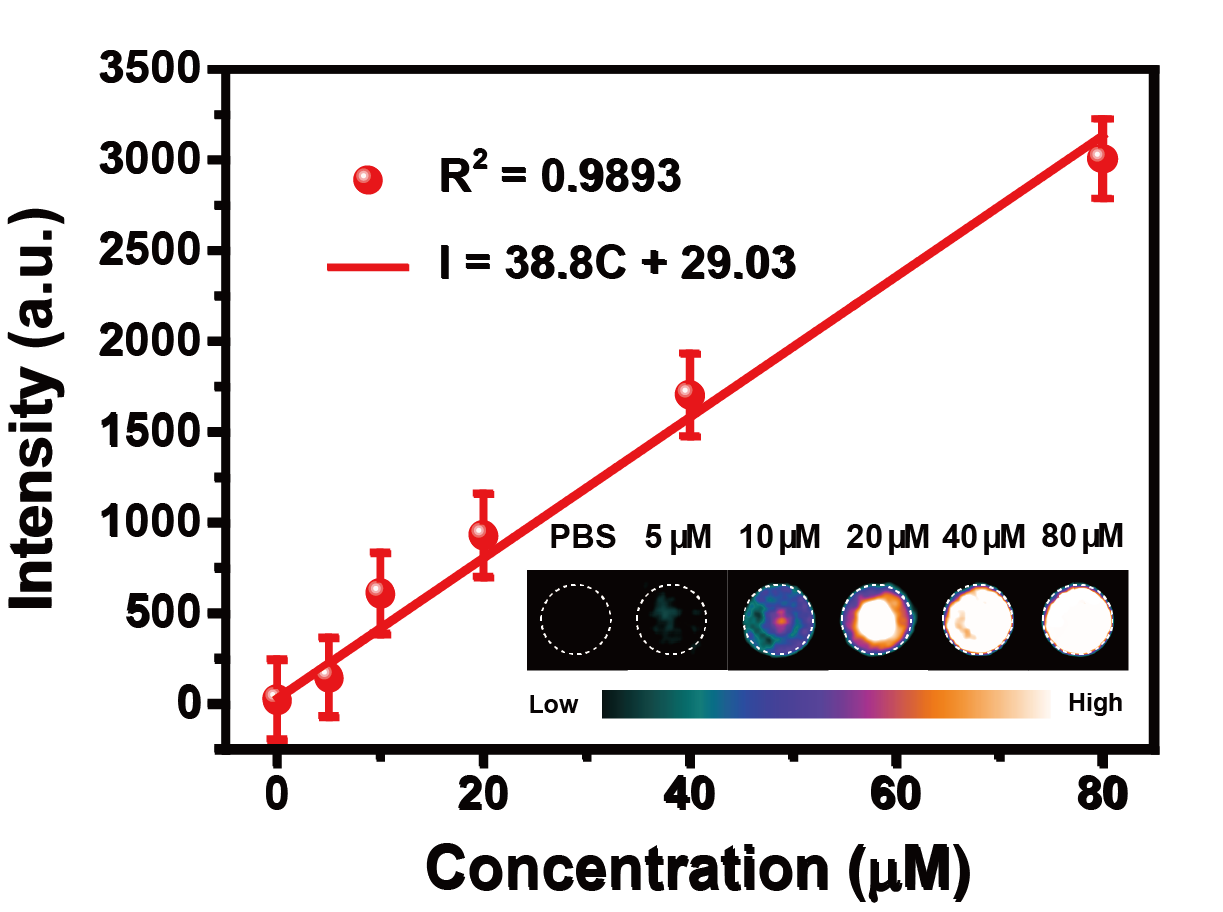


**Fig. S7.** Photoacoustic intensity of **NIR998 NPs** with various concentrations in PBS (pH = 7.4). Inset: photoacoustic intensity images of **NIR998 NPs** with various concentrations in PBS (pH = 7.4) (λ_Ex_ = 808 nm).


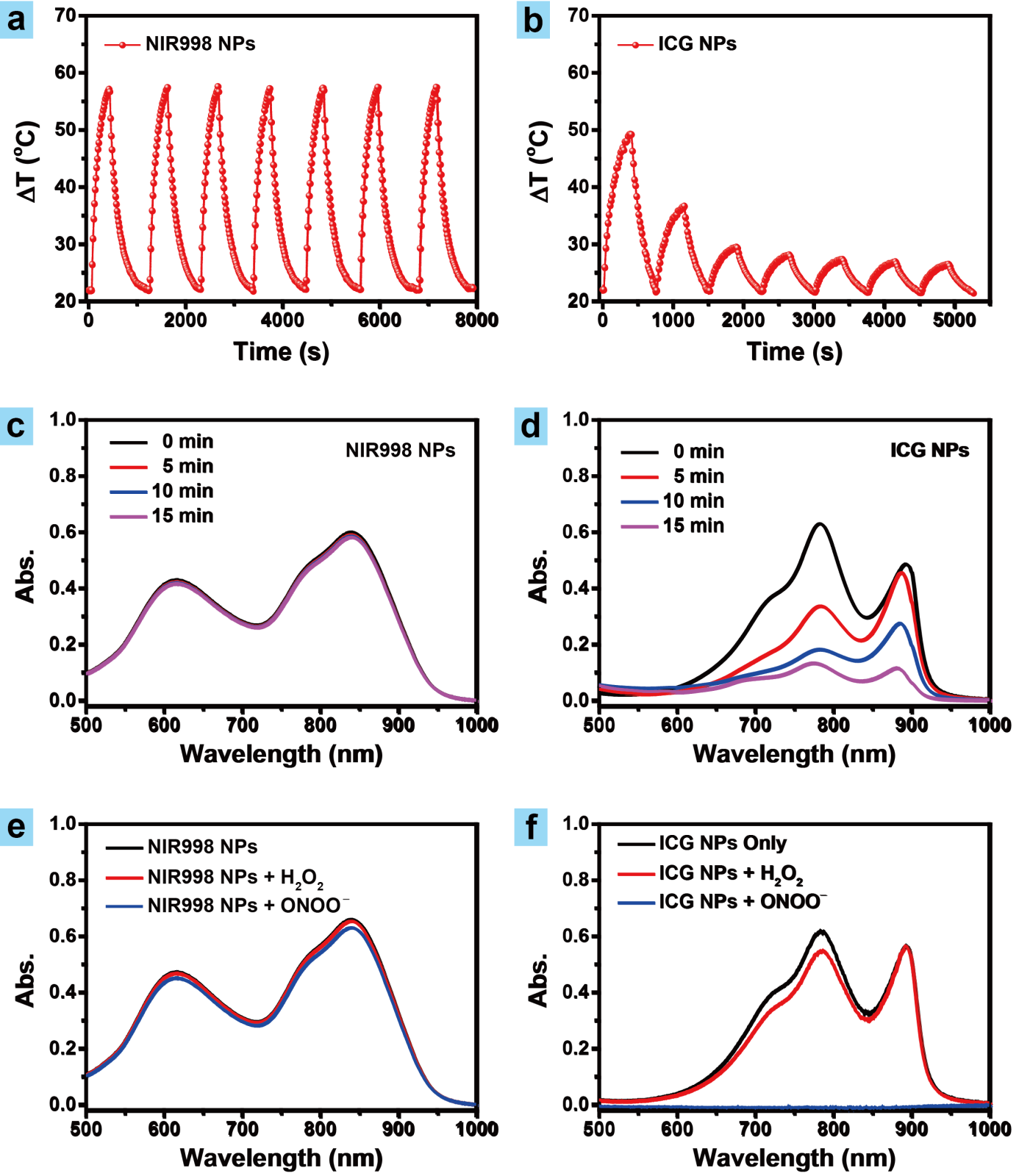


**Fig. S8.** (a) and (b) are photothermal circulation stability of **NIR998 NPs** (20 μM) and **ICG NPs** (20 μM) in PBS (pH = 7.4) under irradiation (808 nm, 0.5 W cm^-2^), respectively. The solutions of samples was irradiated until its temperature reached to plateau. The irradition then stopped. When its temperature decrease to ambient temperature, we then repeated above process for seven times. (c) and (d) are absorption spectra of **NIR998 NPs** (20 μM) and **ICG NPs** (20 μM) in PBS (pH = 7.4) with the absence or presence of H_2_O_2_ (200 μM) or ONOO^−^ (200 μM), respectively.


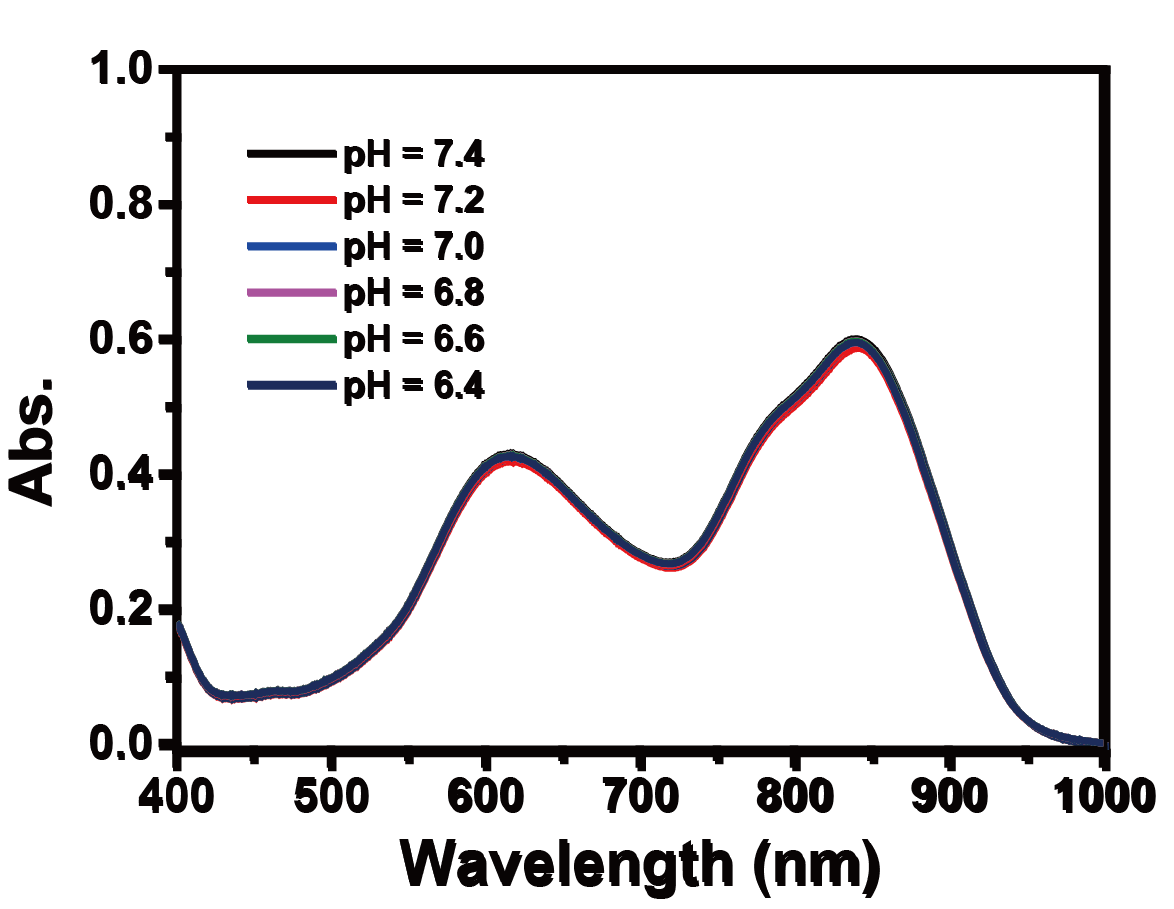


**Fig. S9.** Absorption spectra of **NIR998 NPs** (20 μM) in PBS (pH = 7.4) with various pH (6.4 – 7.4), respectively.


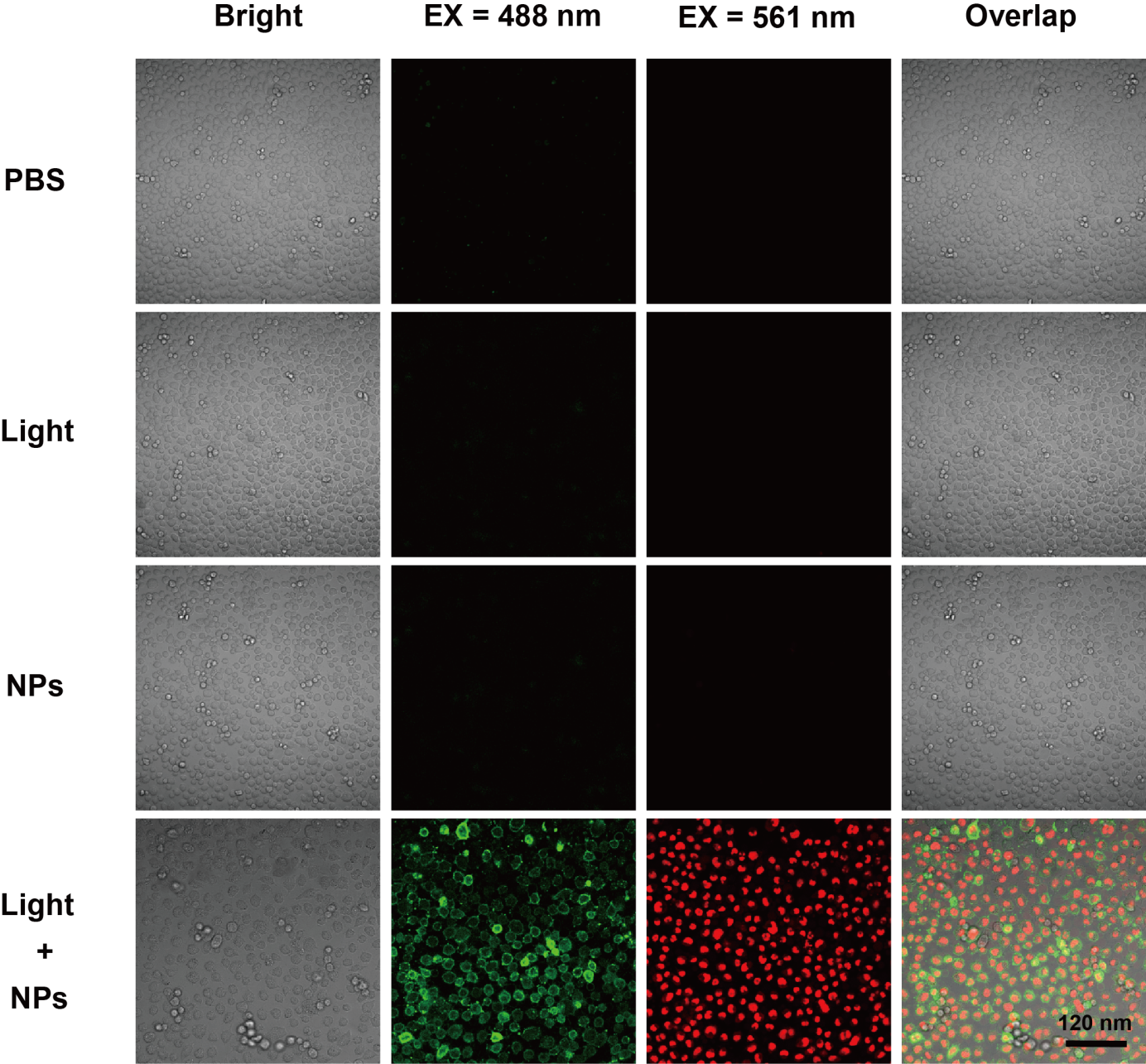


**Fig. S10.** Confocal fluorescence images of SKOV3 cells treated with **NIR998 NPs** (20 μM) plus light irradiation (808 nm, 0.5 W cm^-2^, 6 min), NIR998 NPs only, light irradiation only or PBS only, respectively. Dead cells and apoptotic cells were distinguished by propidium iodide (PI) and Annexin V-FITC, respectively.


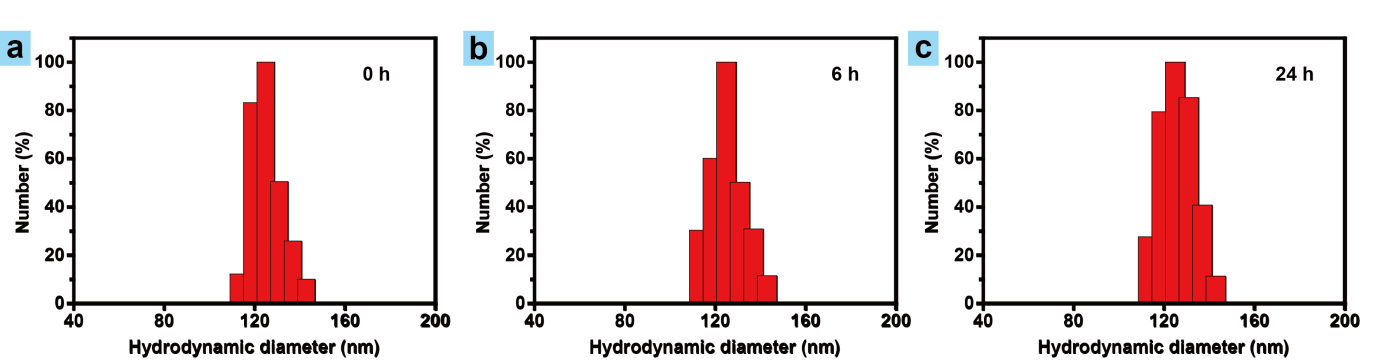


**Fig. S11.** (a), (b), (c) are dynamic light scattering results of **NIR998 NPs** in presence of serum proteins in vitro with time, respectively.


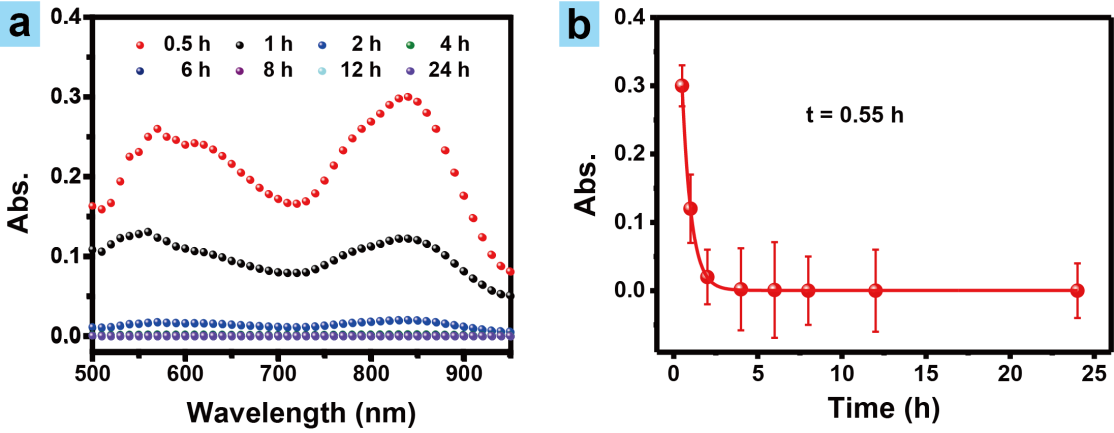


**Fig. S12.** (a) The absorption spectra of **NIR998 NPs** in serum after intravenous injection of **NIR998 NPs** (200 μM, 150 μL) with time. (b) Time-dependent-concentrations curves of **NIR998 NPs** after their intravenous injection in mice.


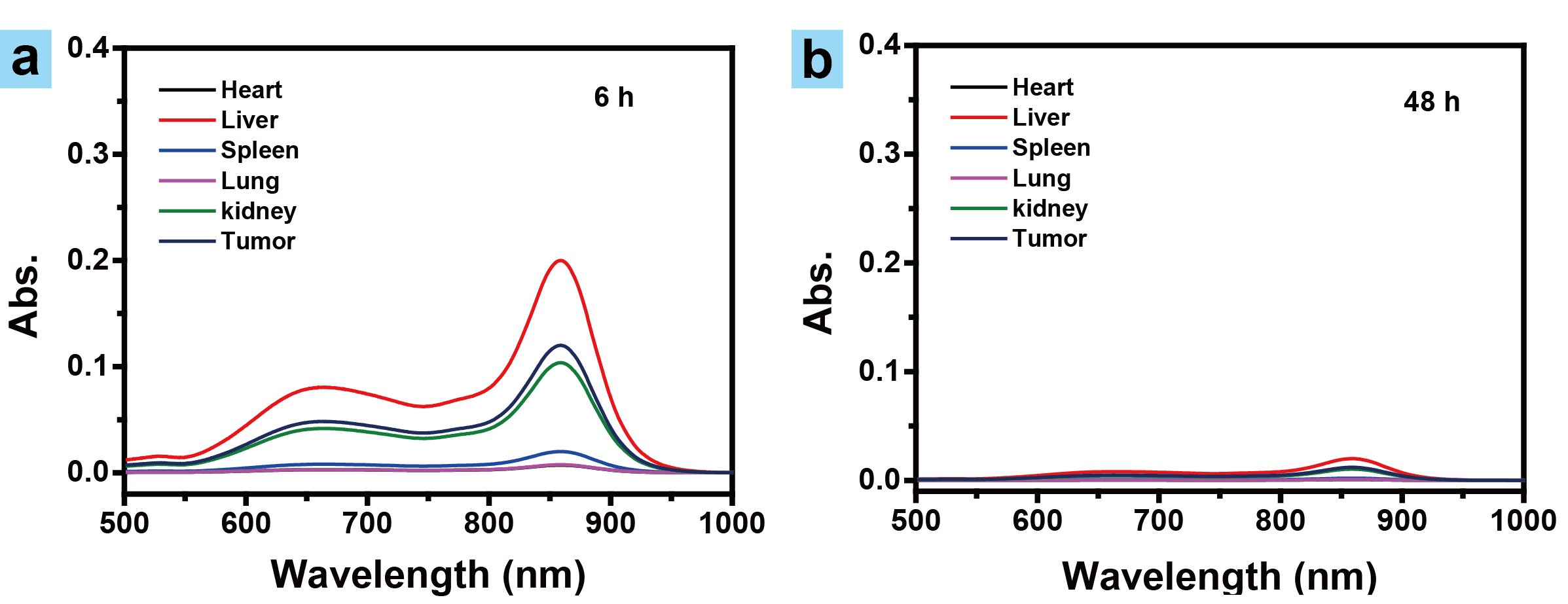


**Fig. S13.** (a) and (b) are the absorption spectra of free **NIR998** obtained from major organs and tumors after intravenous injection of **NIR998 NPs** (200 μM, 150 μL) for 6 h and 48 h, respectively.


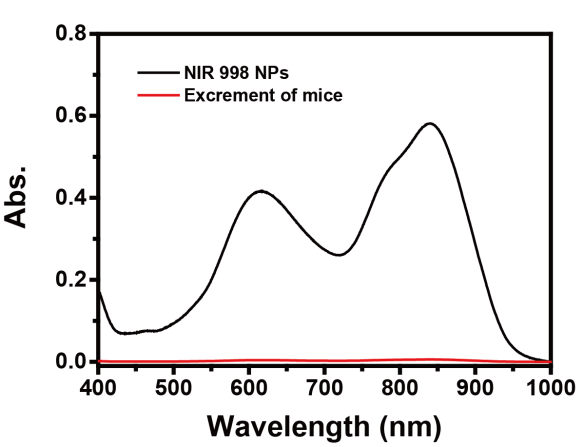


**Fig. S14.** The absorption spectrum of **NIR998** NPs (10^-5^ M) and excrement of mice in PBS (pH = 7.4) after intravenous injection of **NIR998 NPs** (200 μM, 150 μL) for 48 h.


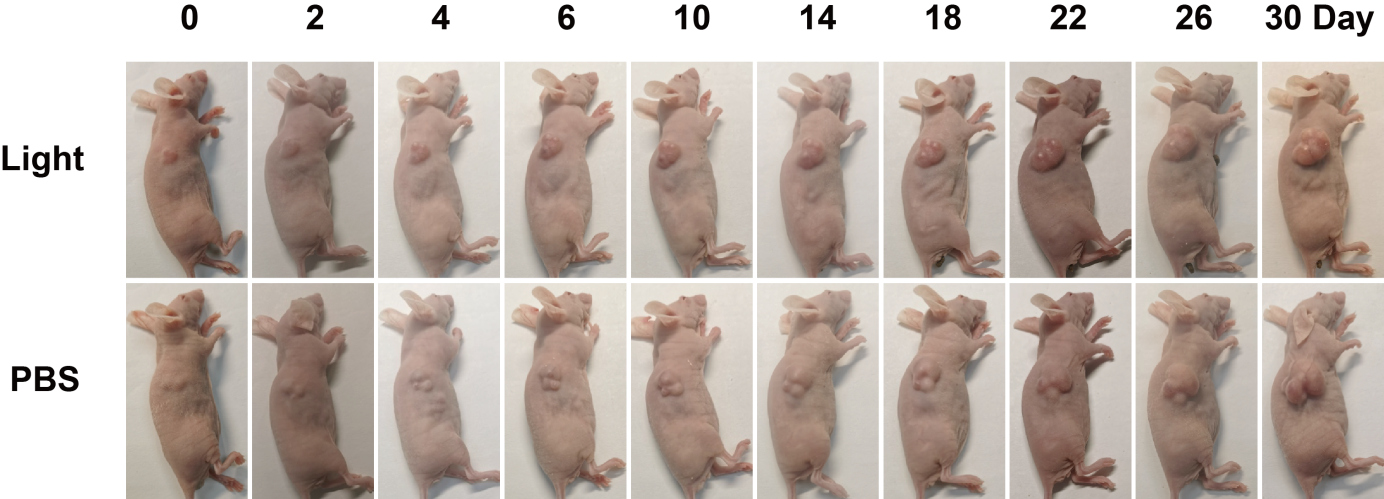


**Fig. S15.** Representative images of mice with SKOV3 tumor under different treatments during tumors therapy, respectively.


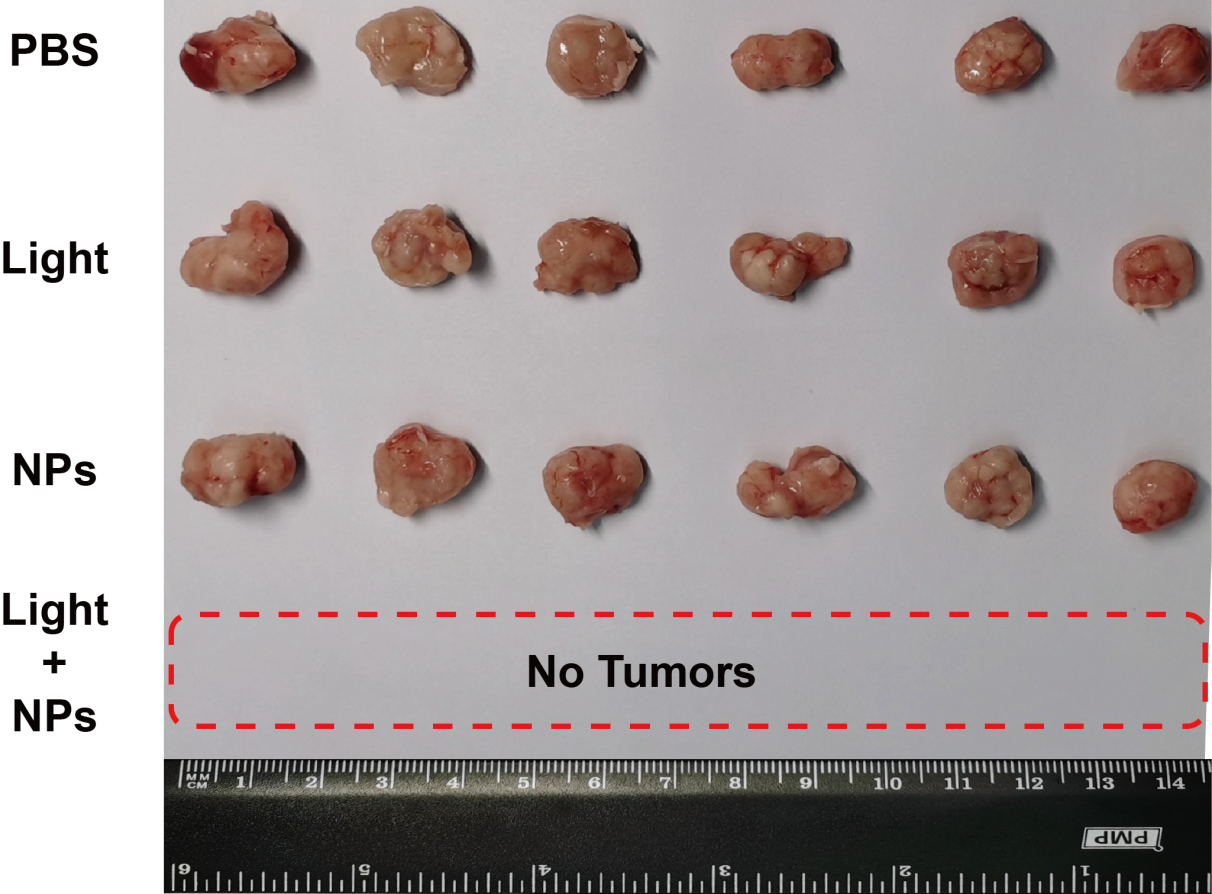


**Fig. S16.** SKOV3 tumors of mice with different treatments after 30 days tumor therapy, respectively.


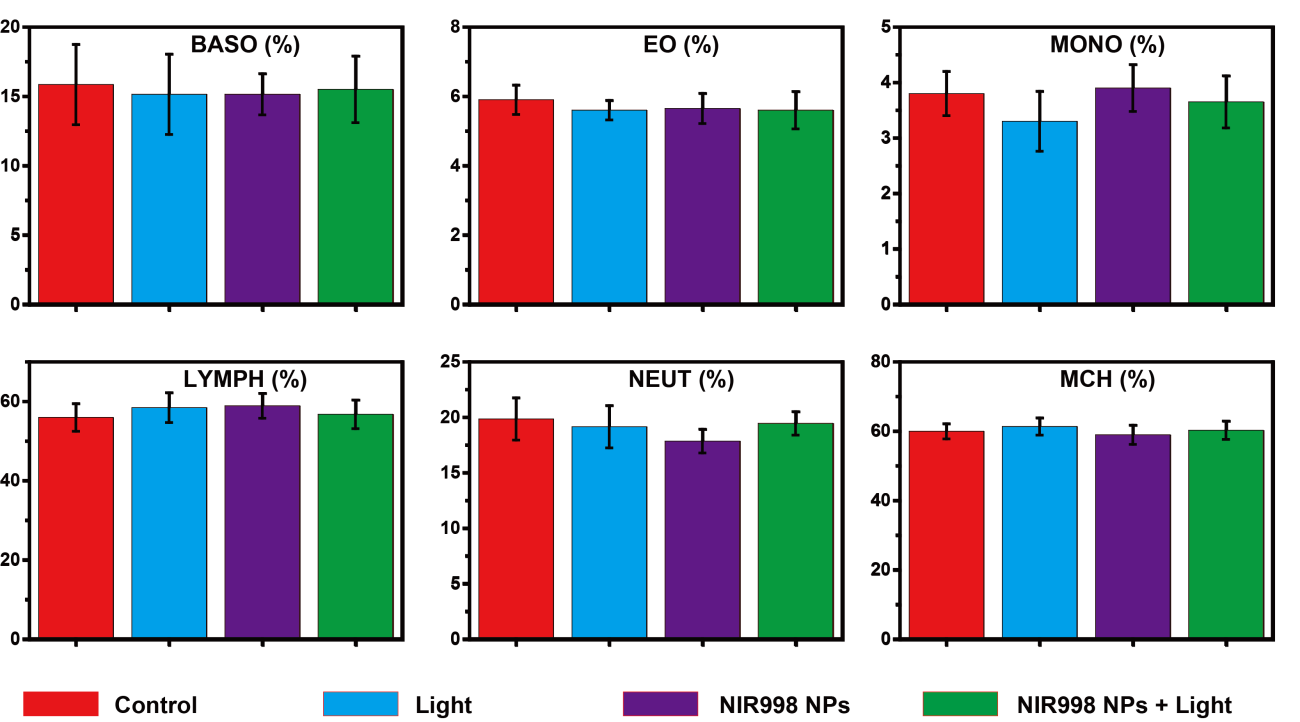


**Fig. S17.** Blood test parameters in terms of basophils (BASO), eosnophilshaem (EO), monocyte (MONO), lymphocyte (LYMPH), neutrophile (NEUT) and mean corpuscular hemoglobinregulation (MCH) of SKOV3 tumor mice treated with **NIR998 NPs** plus light irradiation, **NIR998 NPs**, light irradiation after 30 days treatments, respectively. Healthy mice act as control.

**Part III. Characterization**


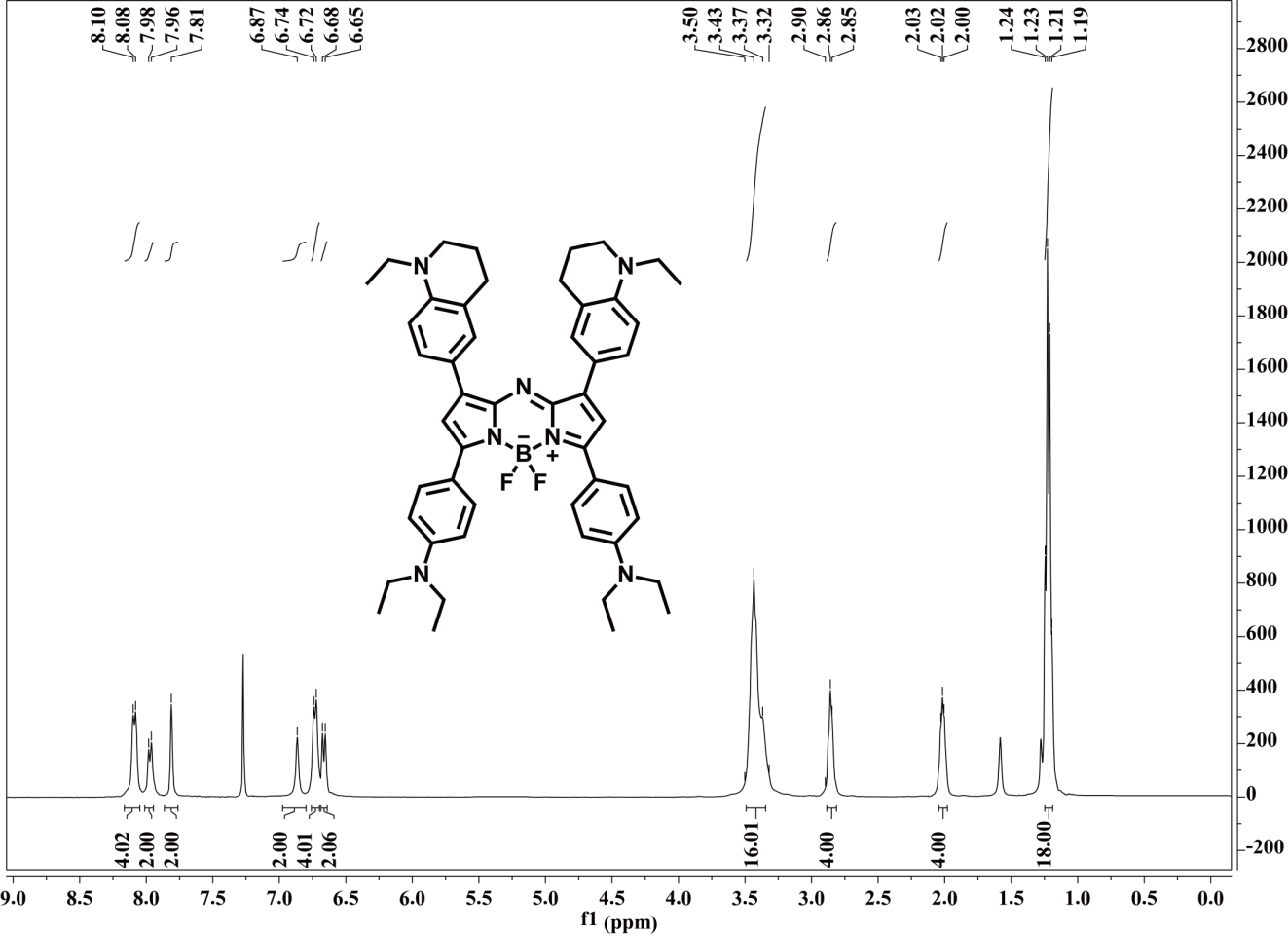


**Fig. S18.** ^1^H-NMR spectrum of **NIR998**.


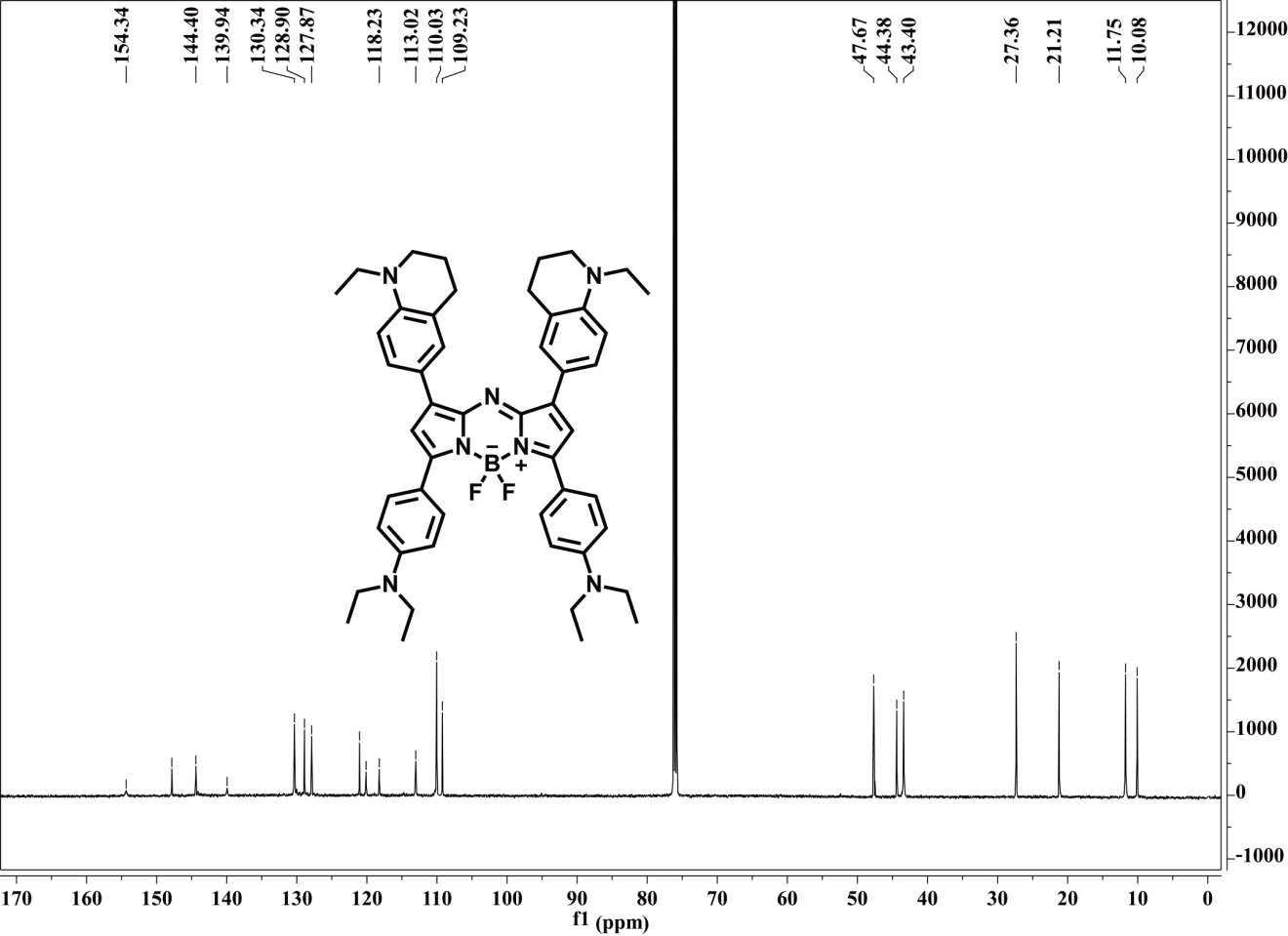


**Fig. S19.** ^13^C-NMR spectrum of **NIR998**.


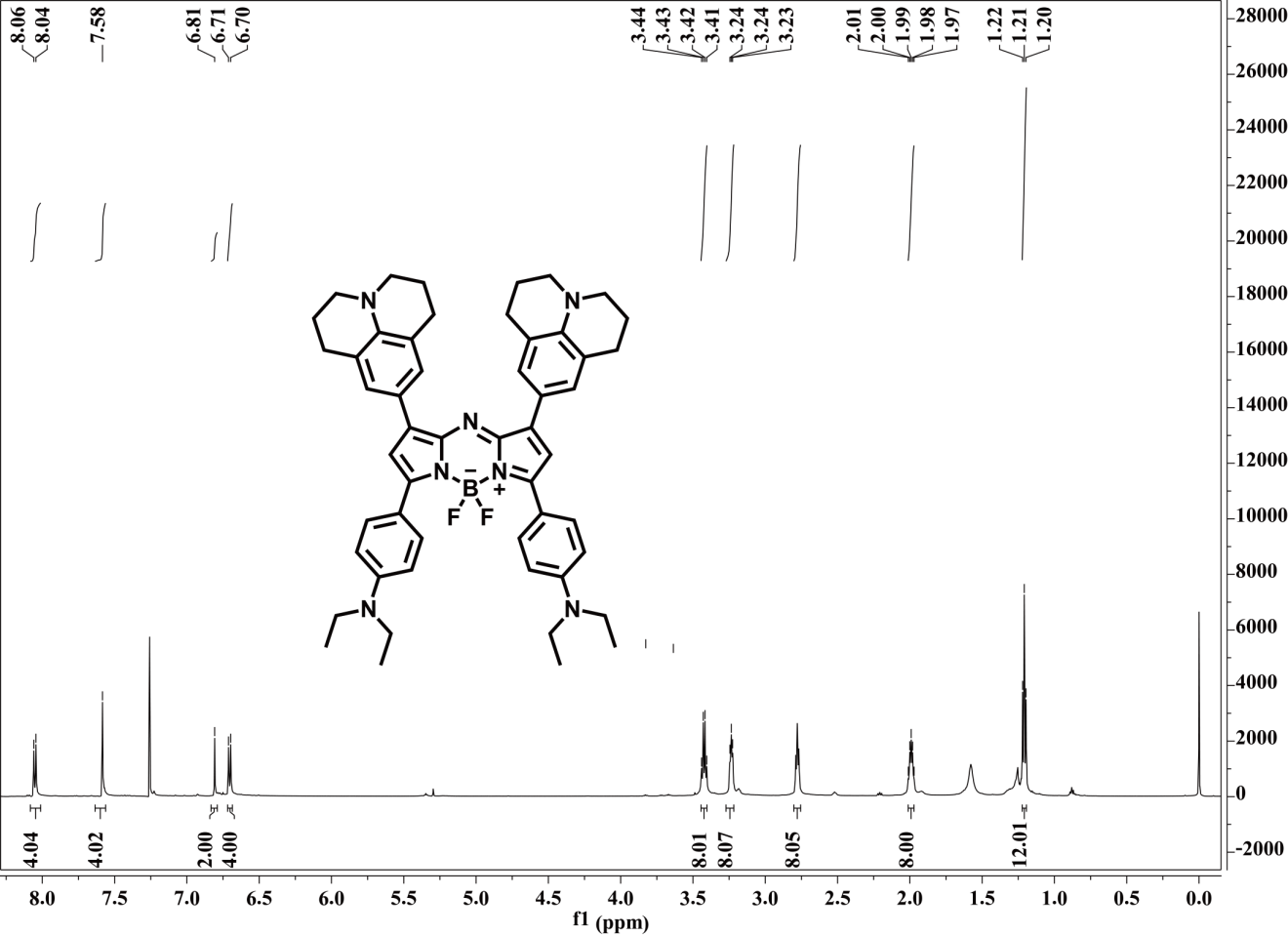


**Fig. S20.** ^1^H-NMR spectrum of **NIR1028**.


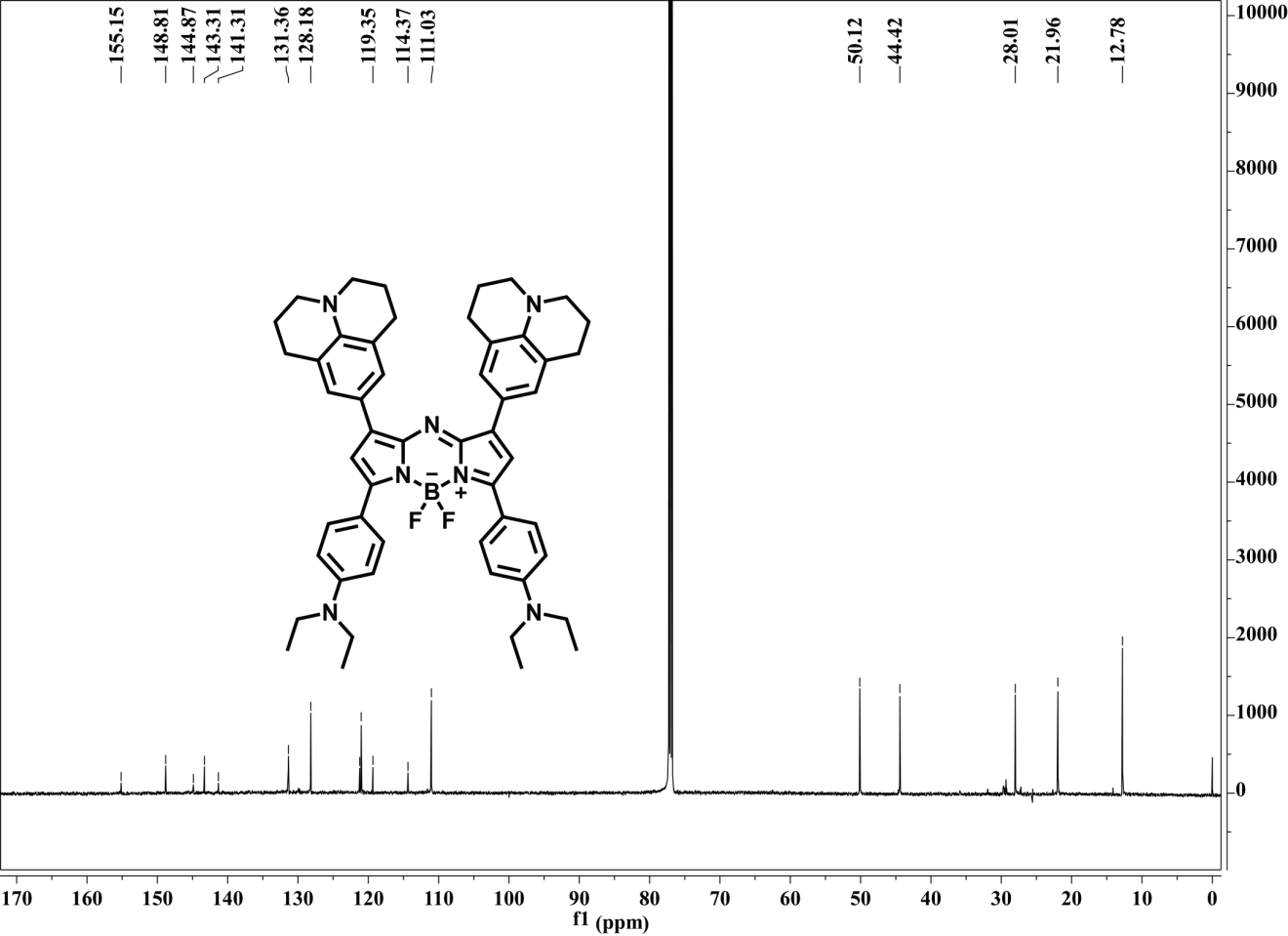


**Fig. S21.** ^13^C-NMR spectrum of **NIR1028**.


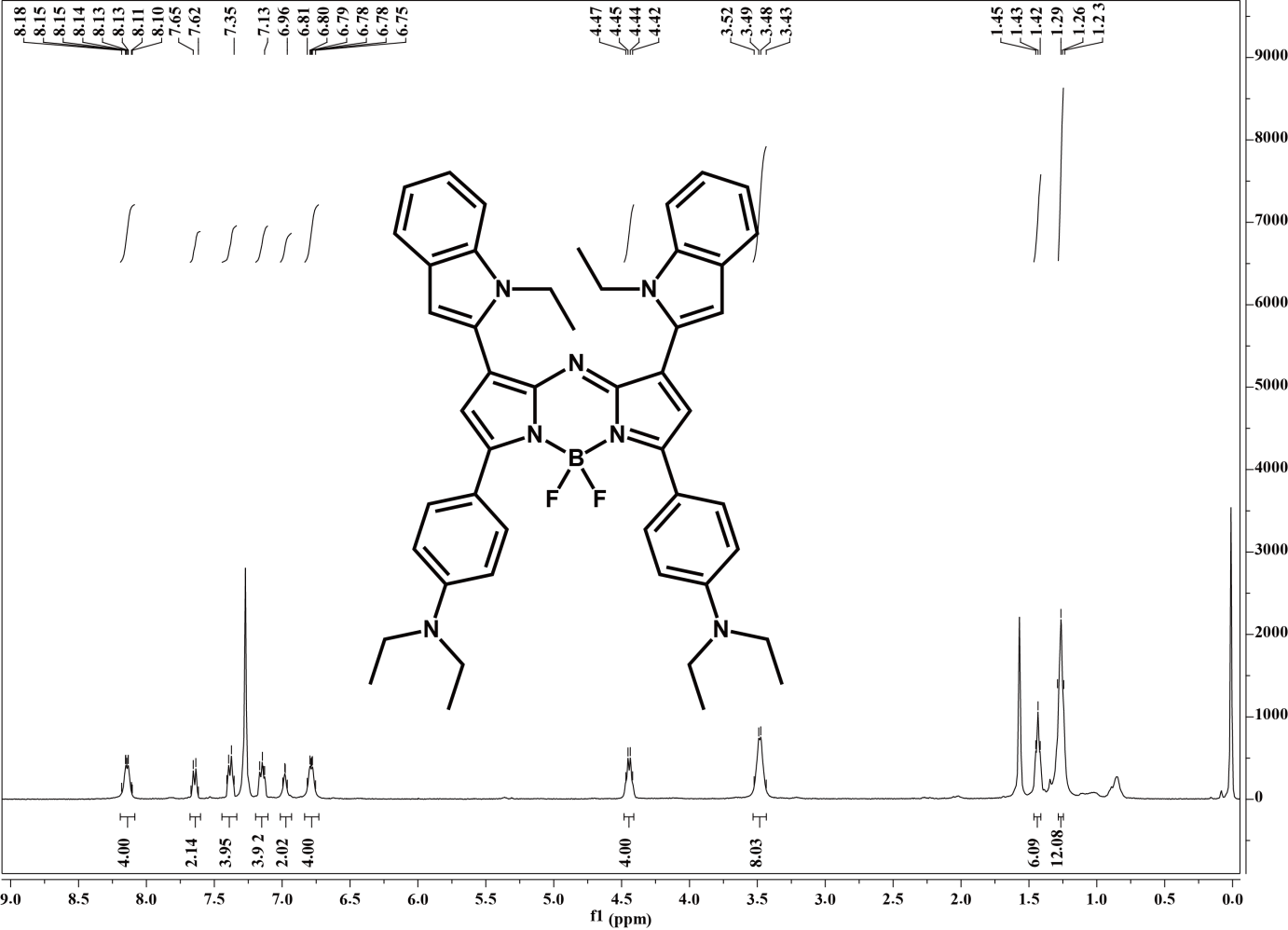


**Fig. S22.** ^1^H-NMR spectrum of **NIR980**.


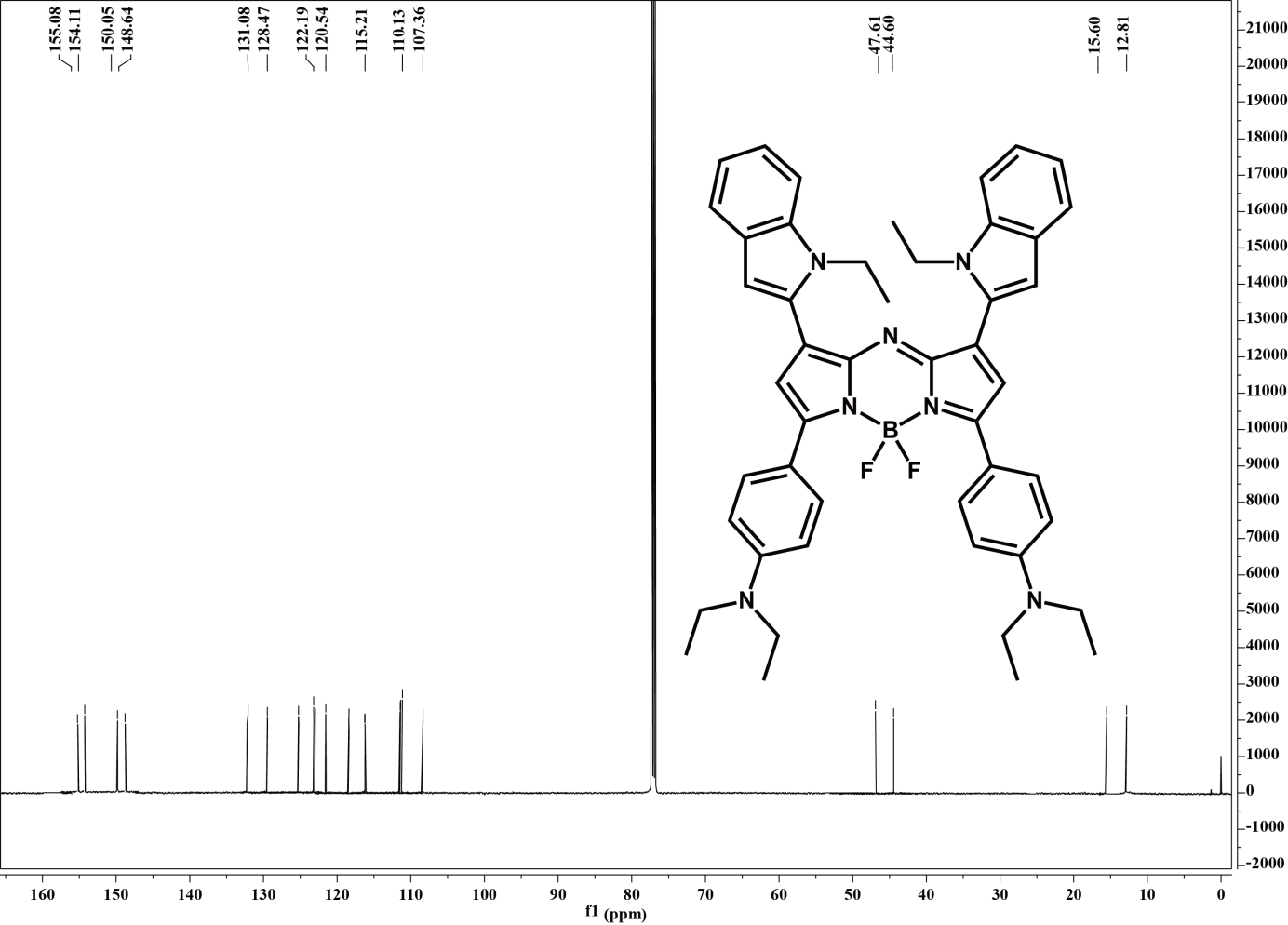


**Fig. S23.** ^13^C-NMR spectrum of **NIR980**.


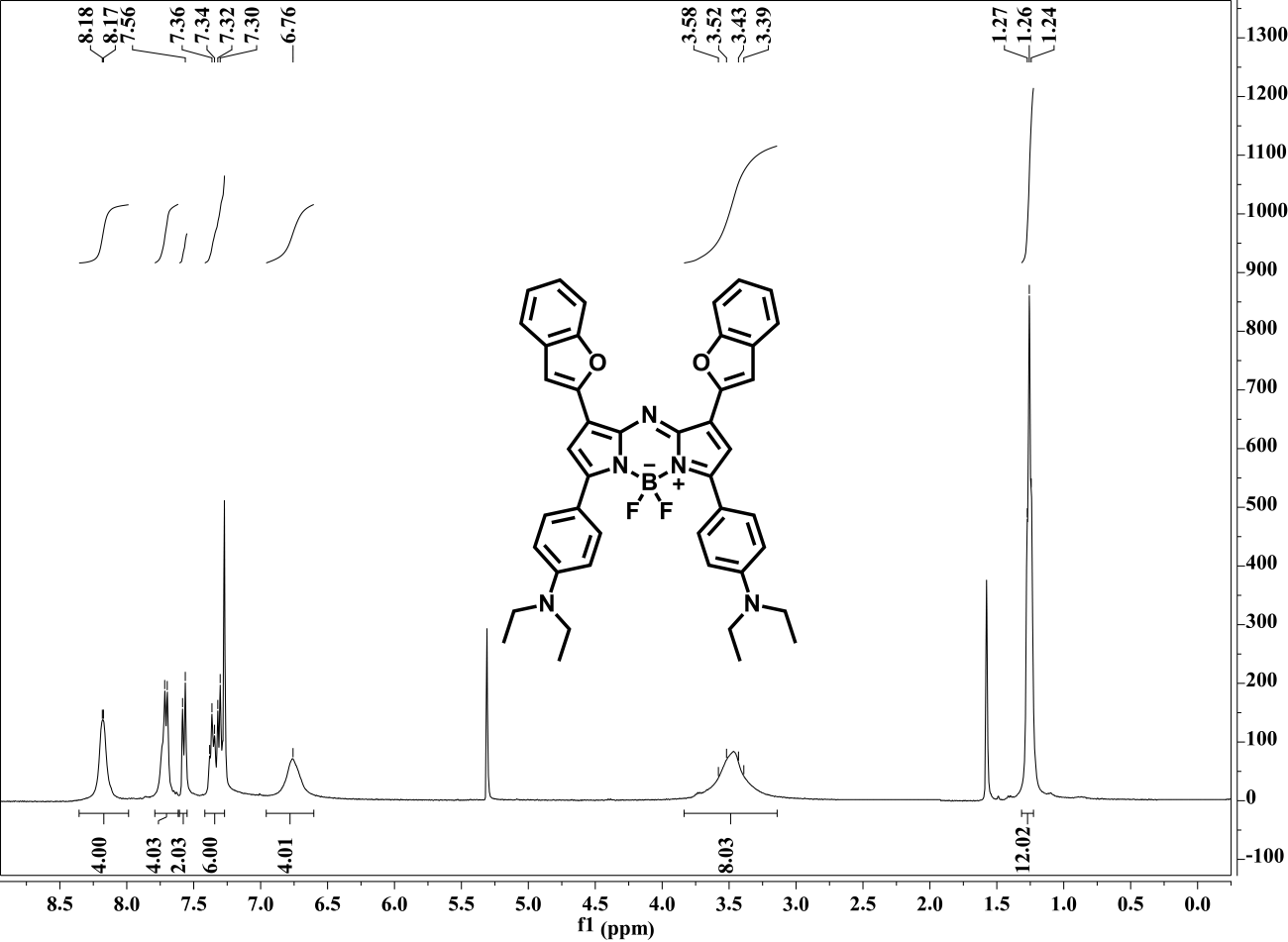


**Fig. S24.** ^1^H-NMR spectrum of **NIR1030**.


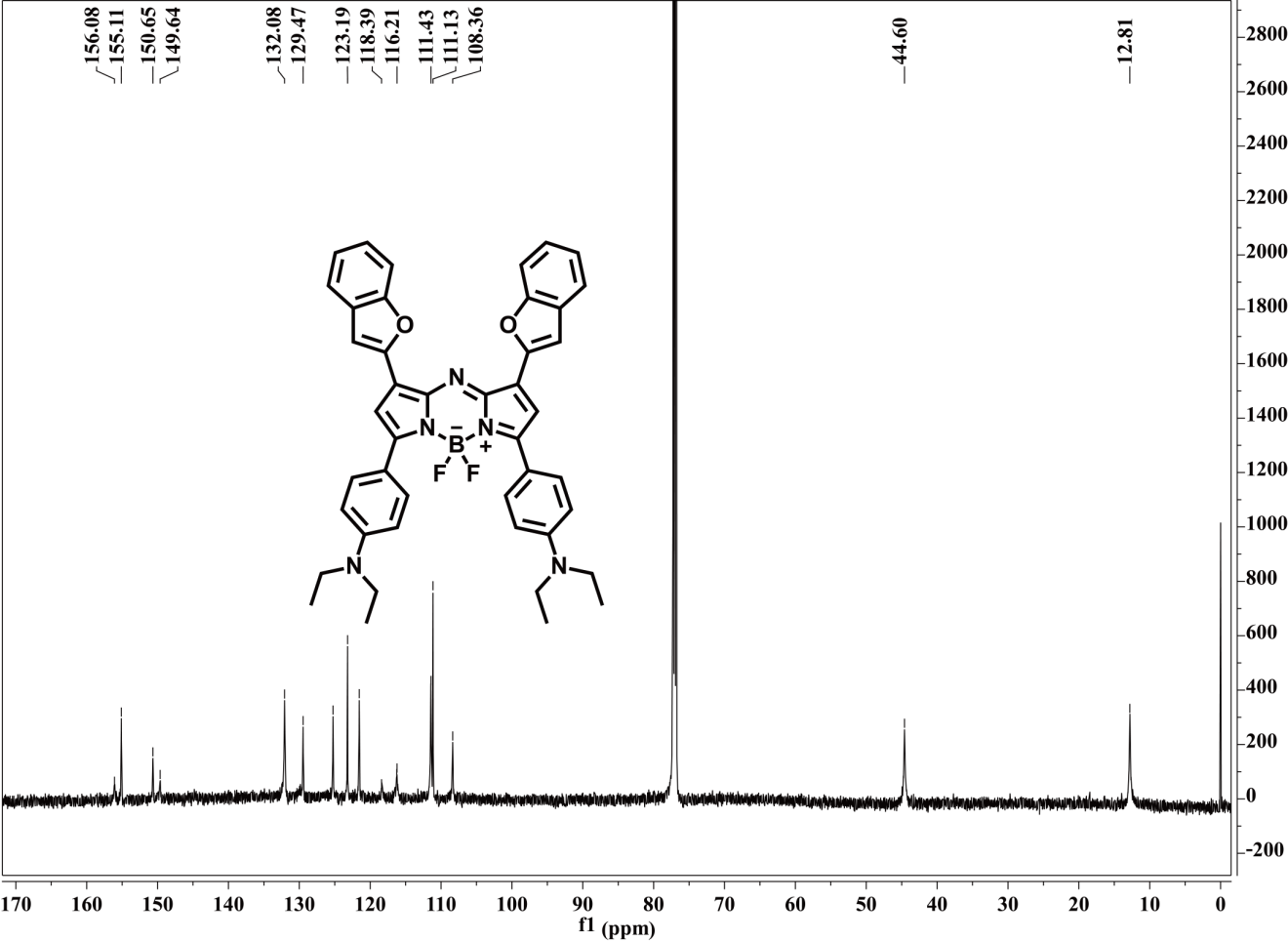


**Fig. S25.** ^13^C-NMR spectrum of **NIR1030**.


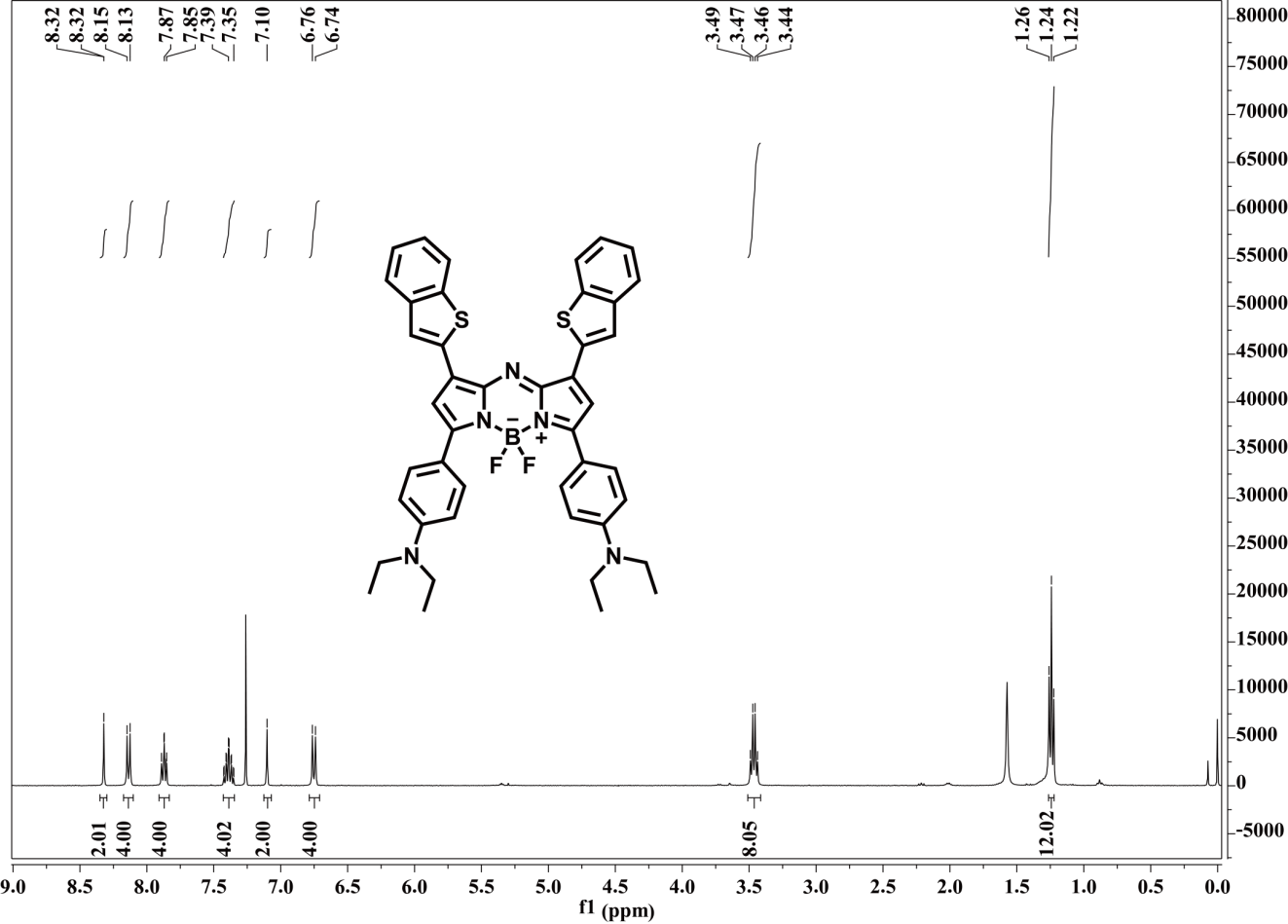


**Fig. S26.** ^1^H-NMR spectrum of **NIR1028-S**.

**
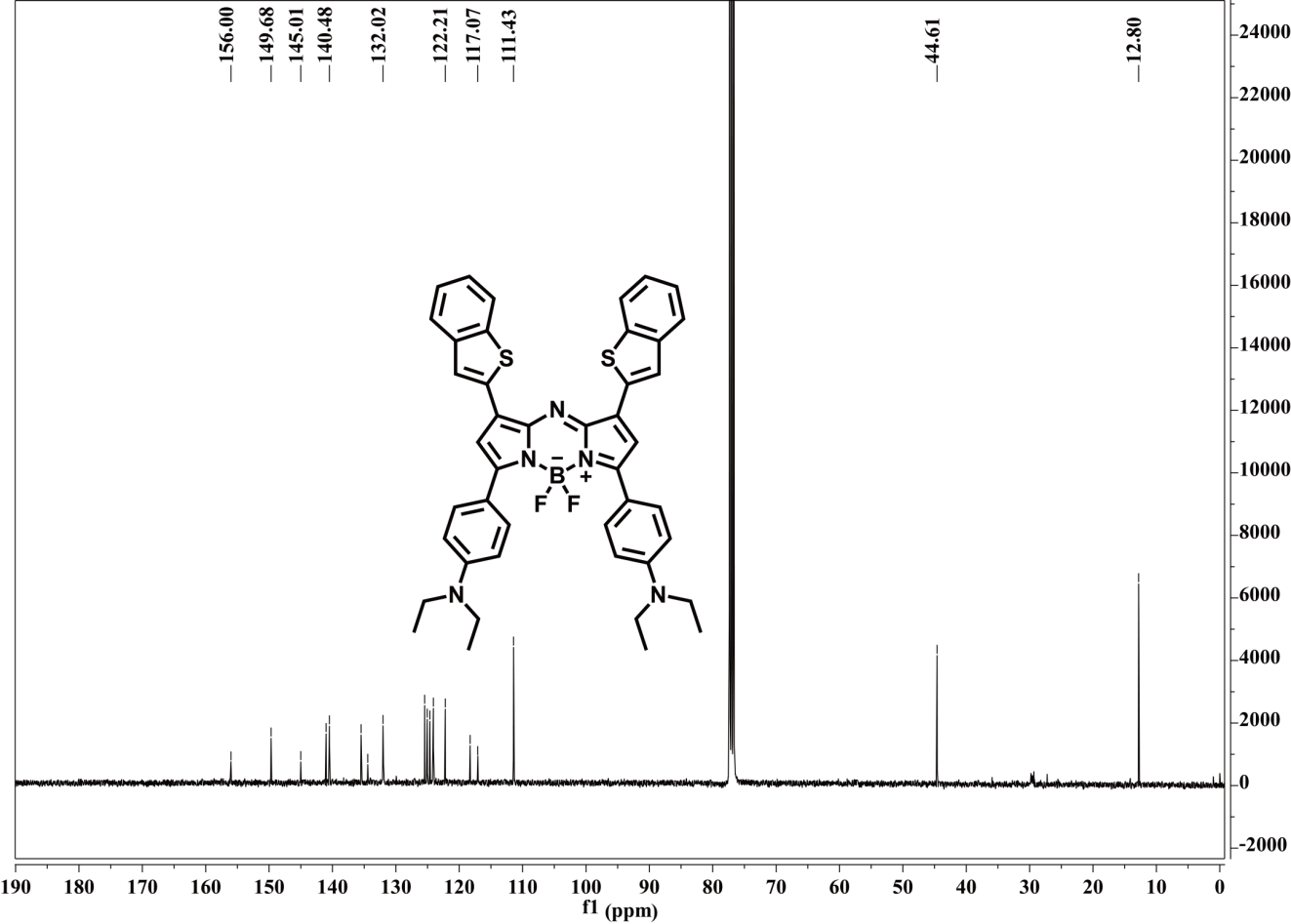
**

**Fig. S27.** ^13^C-NMR spectrum of **NIR1028-S**.


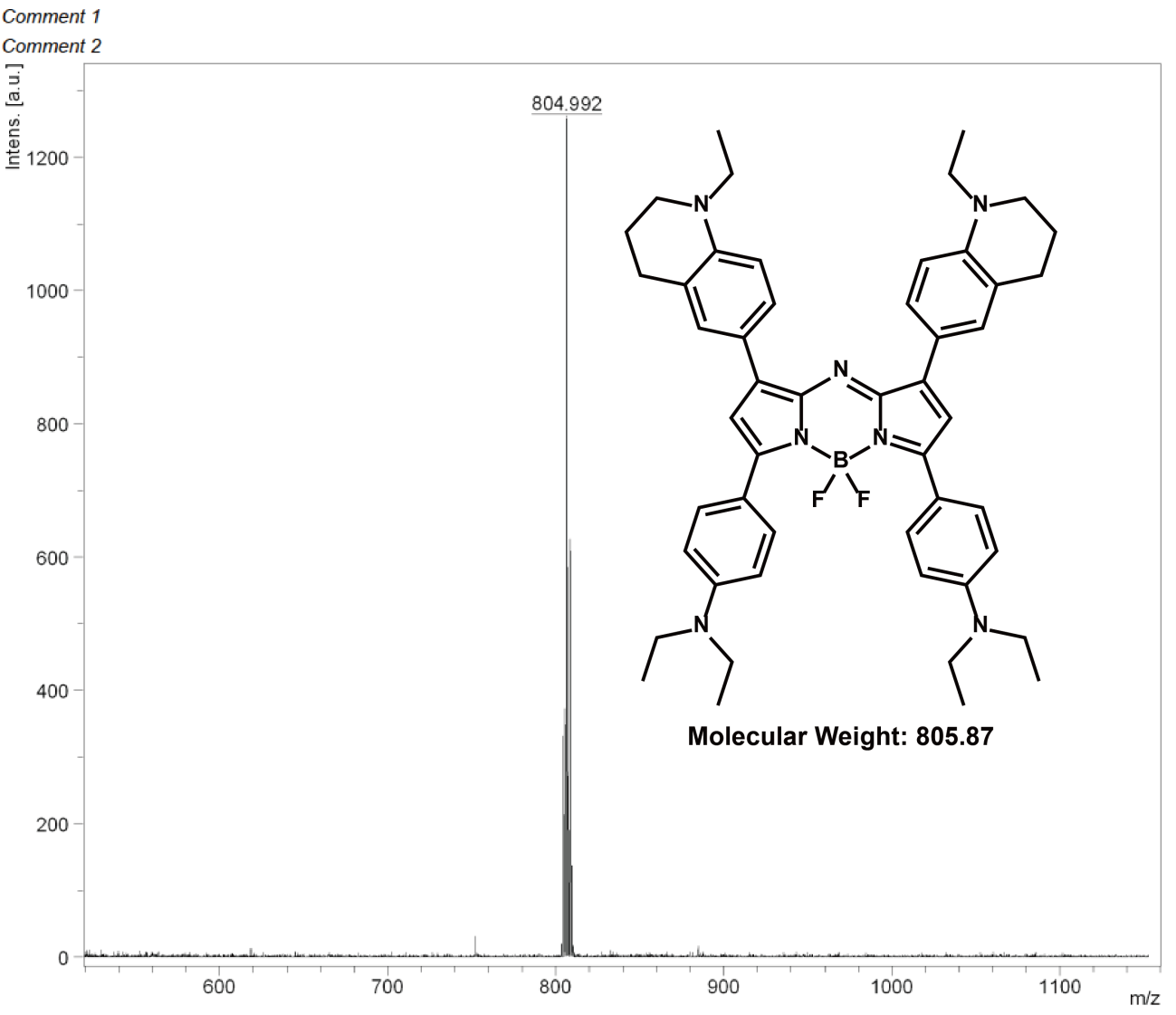


**Fig. S28.** MALDI-TOF-MS spectrum of **NIR998**.


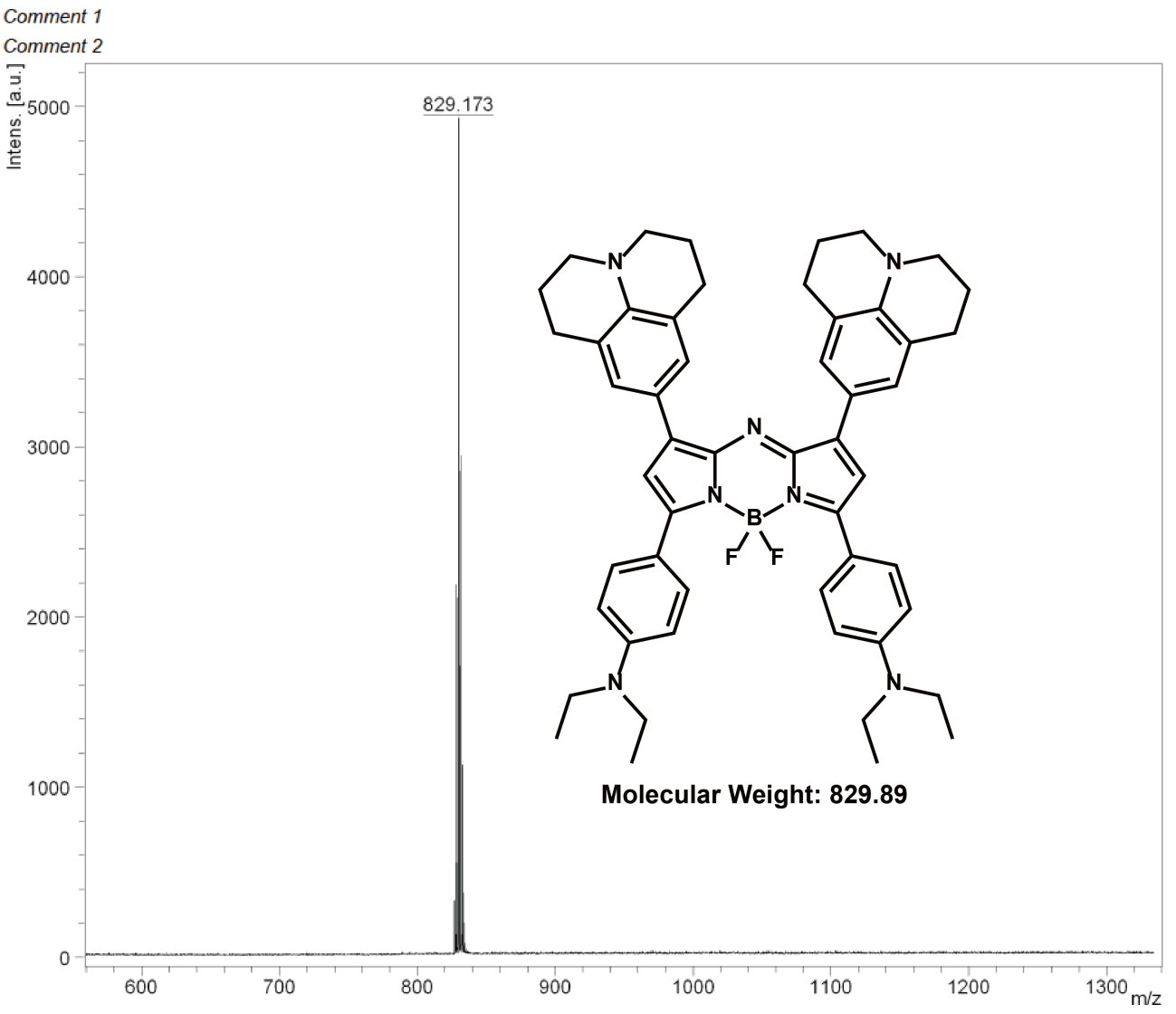


**Fig. S29.** MALDI-TOF-MS spectrum of **NIR1028**.


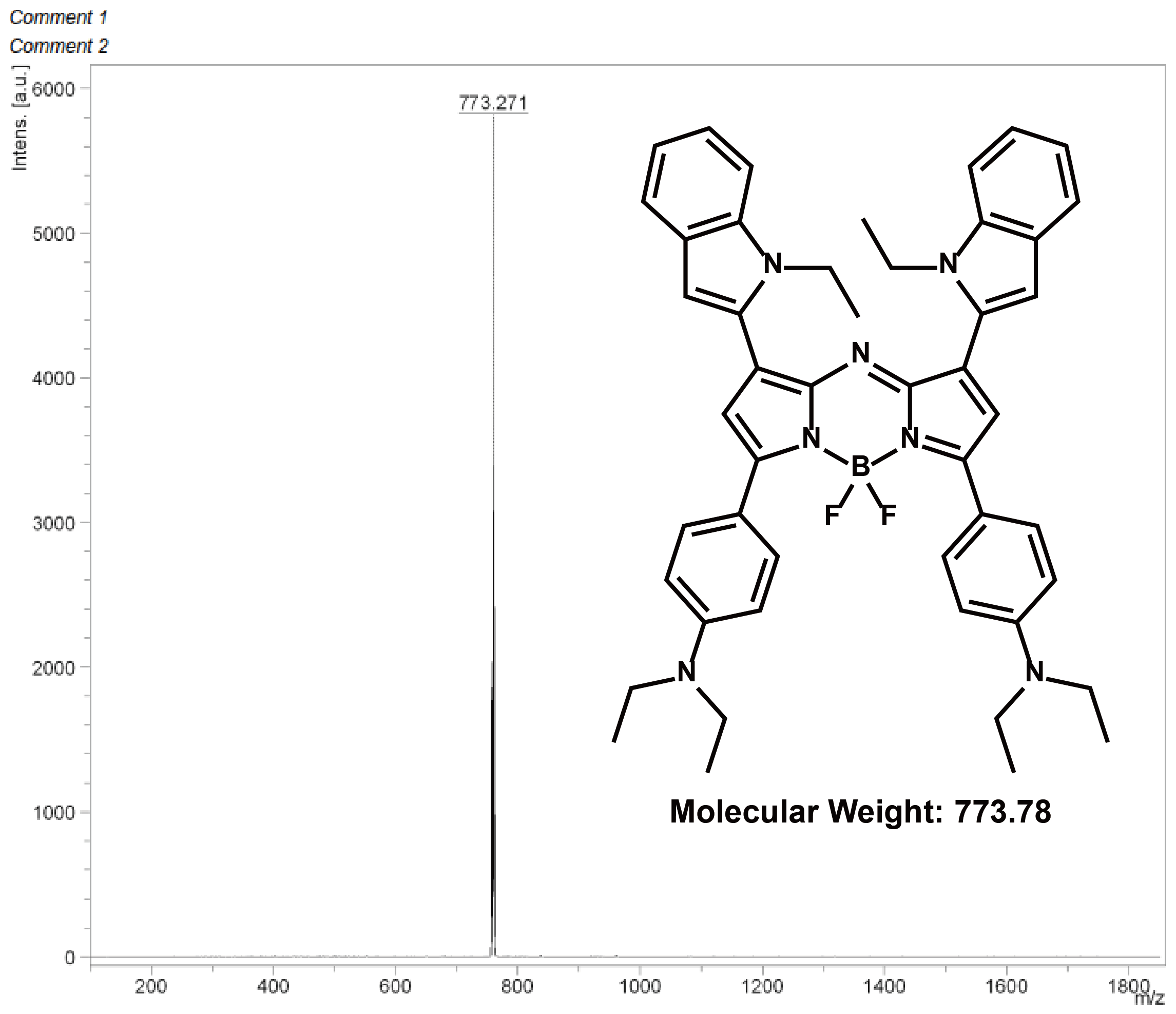


**Fig. S30.** MALDI-TOF-MS spectrum of **NIR980**.


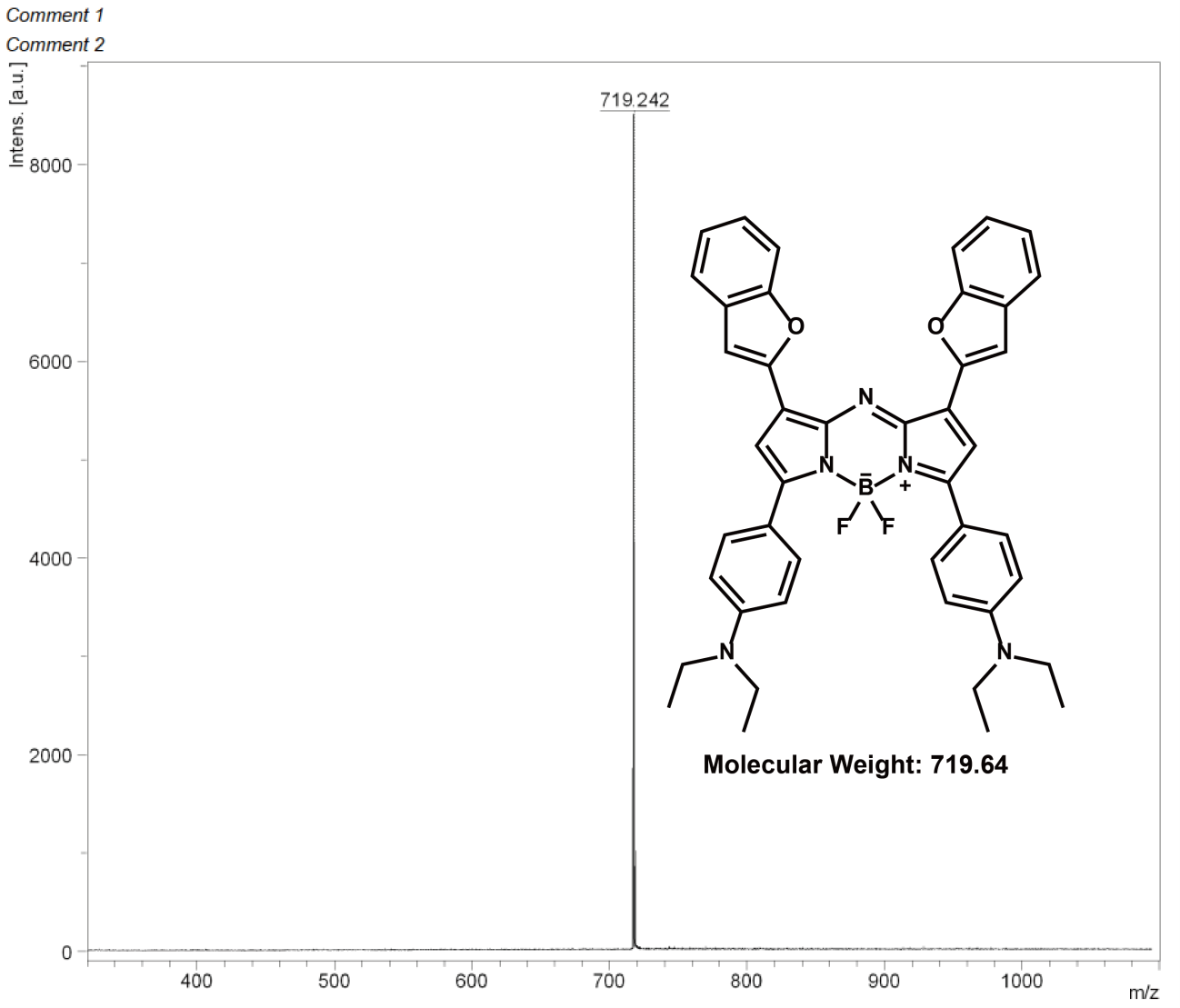


**Fig. S31.** MALDI-TOF-MS spectrum of **NIR1030**.


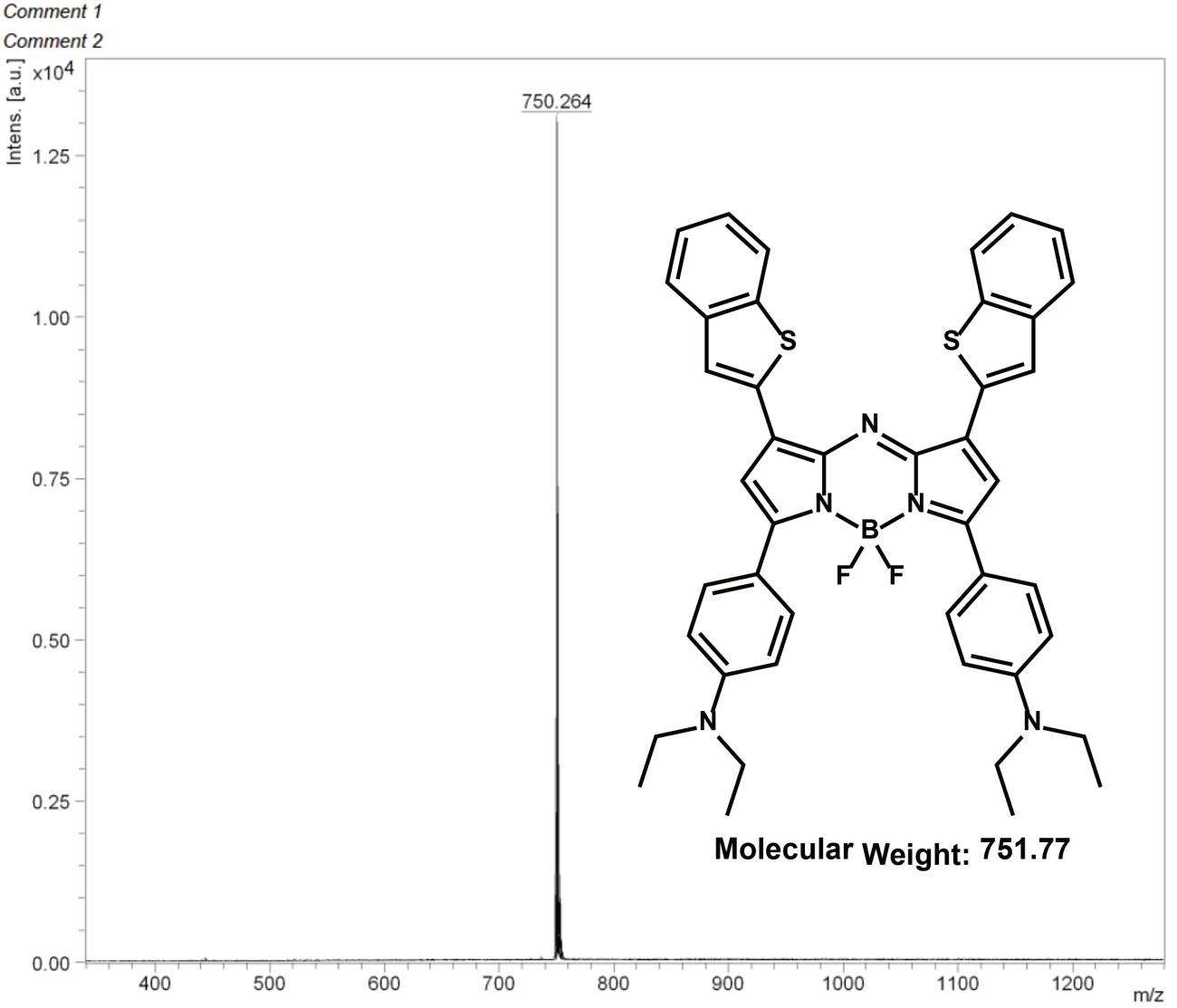


**Fig. S32.** MALDI-TOF-MS spectrum of **NIR1028-S**.
